# Fate of Sc-Ion Interaction With Water: A Computational Study to Address Splitting Water Versus Solvating Sc Ion

**DOI:** 10.3389/fchem.2021.738852

**Published:** 2021-10-18

**Authors:** Nandan Kumar, Y. Bhargav Kumar, Himakshi Sarma, G. Narahari Sastry

**Affiliations:** ^1^ Centre for Molecular Modelling, CSIR-Indian Institute of Chemical Technology, Hyderabad, India; ^2^ Academy of Scientific and Innovative Research (AcSIR), Ghaziabad, India; ^3^ Advanced Computation and Data Sciences Division, CSIR-North East Institute of Science and Technology, Jorhat, India

**Keywords:** metal cation–water interaction, binding energy, electron density, laplacian of electron density, energy decomposition analysis

## Abstract

An exhaustive study of Sc-ion interaction with water molecules in all its possible oxidation and spin states has been carried out to delineate the relative propensity of Sc ions toward solvation and water splitting. Potential energy surface analysis of the Sc-ion reaction with water molecules, topological analysis of bonds, and the effect of sequential solvation up to 6 water molecules have been examined. Calculated values showed good agreement with the available experimental results. Close-shell systems such as singlet mono- and tricationic Sc ions prefer to split the water molecules. In contrast, the open-shell systems such as triplet mono- and doublet dicationic Sc ions prefer to get solvated than split the water molecule. Topological analysis of electron density predicted the Sc^+/2+^–water bond as a noncovalent bond while Sc^3+^–OH_2_, Sc^2+^–OH, and Sc^+^–H bonds as partially covalent in nature. Energy decomposition analysis revealed that Sc ion–water interactions are driven by electrostatic energy followed by polarization energy. The current study reveals that transition metal catalysis can be one of the most effective tools to employ in water splitting, by properly tuning the electrons, spin, and ligands around the catalytic center.

## Introduction

The reaction of bare transition metal ions is of utmost importance, given the role they play in a variety of biological and chemical processes (Williams, 1968; Sundberg et al., 1974; Armentrout et al., 1989; Armentrout, 1991; Hippeli et al., 1999; Mahadevi et al., 2013; Swart et al., 2016; Goodman et al., 2019). However, understanding the chemistry of these ions is a challenging endeavor due to variable oxidation states and multiple spin states (Poli, 1996; Harvey et al., 2003). The reaction of transition metal ions, main-group atomic ions, and lanthanide ions with water molecules is studied using experimental methods (Kauffman et al., 1985; Rosi et al., 1989; Dalleska et al., 1994; Trachtman et al., 1998; Cheng et al., 2007; Mo et al., 2007; Saha and Sastry, 2015a). These studies have been followed by several detailed computational analyses on the nature of metal ions interacting with one, two, or more water molecules (Rao et al., 2008; Neela et al., 2013a; Neela et al., 2013b; Mahadevi and Sastry, 2014; Magnera et al., 1989; Marinelli et al., 1989; Tilson et al., 1991; Irigoras et al., 1998, Irigoras et al., 1999a; Irigoras et al., 2000; Chiodo et al., 2004; Meng et al., 2012; Sharma et al., 2016). These studies have provided valuable insights into the coordination preferences of the corresponding metal ions. Solvation of transition and nontransition metal ions has been investigated by employing high levels of theory for the calculation of the accurate structure and strength of the small cation–water clusters (Russo and Sicilia, 2001; Neela et al., 2010; Sharma et al., 2011; Umadevi et al., 2011; Mahadevi et al., 2014; Umadevi et al., 2014; Sharma et al., 2015; Saha and Sastry, 2015b; Sharma et al., 2016). Clemmer et al., 1993 reported that early first-row transition metal ions are more reactive than their oxides, while the oxides are more reactive than the metal ions for late first-row transition metal ions. Sharma et al., 2015 and Kumar et al., 2021 have reported that the interaction of alkali and alkaline earth metal ions (except Li^+^) with water molecules are primarily electrostatic driven. These studies have provided valuable insights into the behavior and chemistry of these ions in the solvent and gas phase. Metal ion–mediated reactions are studied exhaustively for the examination of properties of metal ions, and in a number of cases, the products occurring at intermediate steps along the reaction path are by themselves very interesting species with unique chemical properties. In this work, a rigorous theoretical investigation of Sc ion interaction with water molecules in all the possible oxidation and spin states has been carried out. A metal ion interacts with water, essentially leading to these possibilities, 1) hydrate: M^z+^(OH_2_)_n_, 2) hydrolyse: H–M^z+^–OH (H_2_O)_n-1_ or H_2_–M^z+^–(OH)_2_ (H_2_O)_n-2_, and 3) dehydrogenate: M^z+^O + H_2_. Additionally, the metal ion may lead to the formation of 4) metal hydroxide: M^(z−1)+^OH or 5) metal hydride: M^(z−1)+^H. Special emphasis is given on understanding the preference of the Sc ion in its variable oxidation states and its corresponding spin states either to split the water molecule by breaking the covalently bonded O–H electron pair or to get solvated by interacting with the lone pair on the oxygen atom. The goal of studying the hydrated ions is to relate the intrinsic physical properties of bare ions to those in an aqueous solution. Understanding the chemistry of a bare metal ion interacting with water molecule is interesting in its own right. The fundamental question in this regard is which of the following mechanisms are predominant: 1) solvation of the metal ion or 2) splitting of the water molecule, with the production of species such as metal hydroxide, metal-hydride, and metal oxide. Thus, the question to be addressed in the current study is that when a bare metal ion interacts with a water molecule in the gas phase, does the process involve a noncovalent interaction limiting to a solvation process or involve the rupturing of bonds in the water molecule leading to covalent bonds. Obviously, the chemistry of water splitting is extremely important with wide-ranging application potential in the energy sector, oxygen generation, and other processes involving O–H bond activation. The current study aims to address the question of Sc-ion interaction with water molecules in the gas phase, followed by the study of microsolvation processes involving the first and second solvation shells. From such measurements, the role of the solvent in the metal-ion structure and reactivity can be comprehended. Potential energy surface analysis of Sc-ion reaction with water molecules and the sequential solvation effects are inspected in detail. However, the quantum theory of atoms in molecules (QTAIM) is used to explore the nature of the interaction, and localized molecular orbital energy decomposition analysis (LMOEDA) is used to examine the contribution of energy components into the metal ion–water interaction.

## Computational Details

**FIGURE 1:**
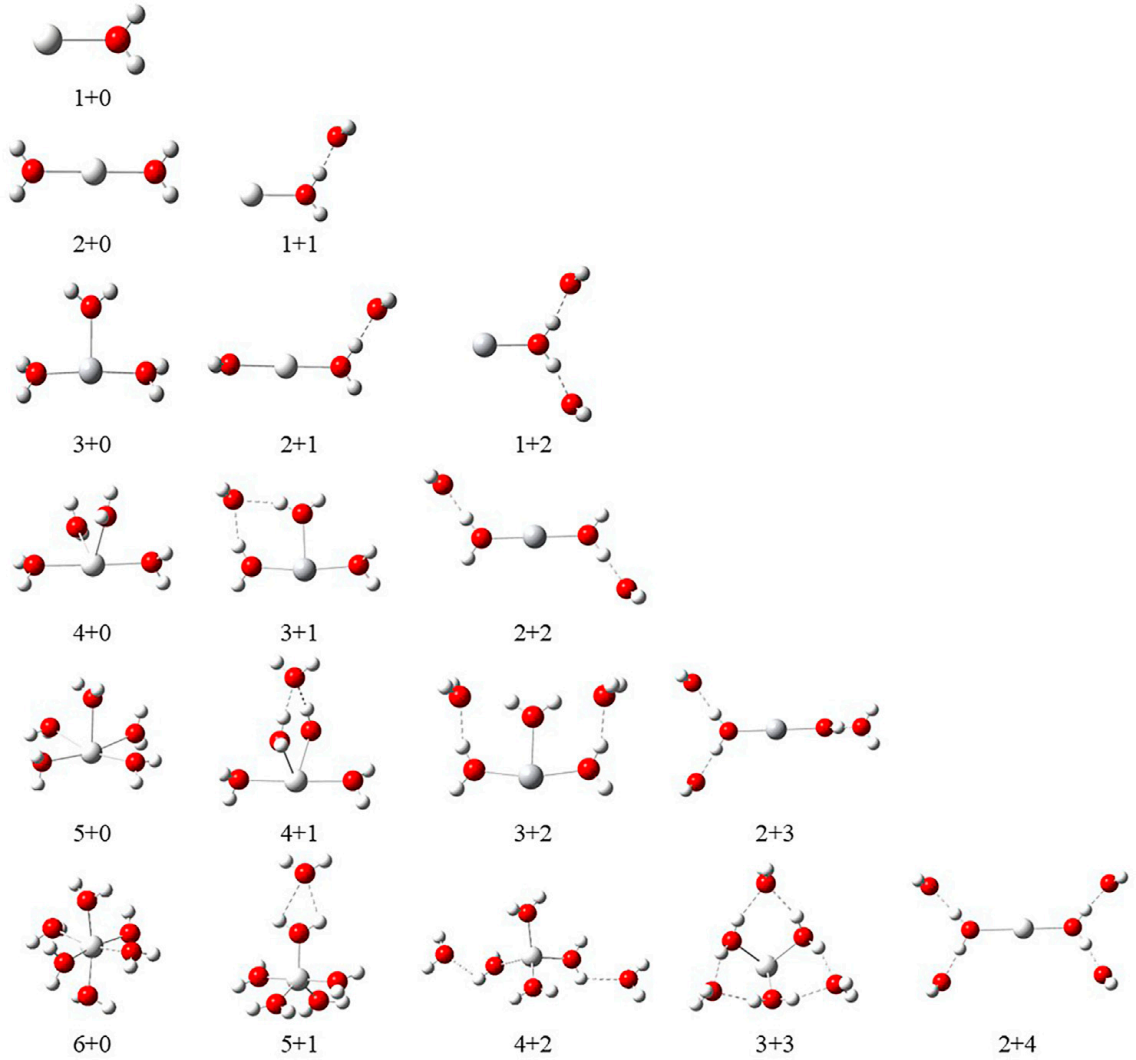
Schematic representation of Sc^z+^(OH_2_)_n_, where z = 1–3 and n = 1–6, complexes and their nomenclature by a label of X + Y (X = number of water molecules in the first solvation shell and Y = number of water molecules in the second solvation shell).

Geometry optimization and frequency calculations were carried out at MP2/6-31G* and B3LYP/6-31G* levels of theory. The energy status of the bare Sc^+^ ion interacting with water was comprehensively examined at 48 levels of theory by making combinations of six methods, that is, HF, PBEPBE, B3LYP, M06, MP2, and CCSD(T), with eight different basis sets, that is, LanL2DZ, DGDZVP, Def2TZVP, 6-31G*, 6-311+G*, 6-311G**, cc-pVTZ, and aug-cc-pVTZ. Subsequently, the best combinations were selected to delineate the interaction of the Sc ion with water molecules. Interaction energy (IE) and sequential binding energy $\left({\text{ΔE}}_{\text{seq}}\right)$ were calculated using Eqs. 1 and 2, respectively. The schematic depiction of the representative structure of Sc(OH_2_)_n_, where n = 1–6, ion complexes and their nomenclature are shown in Figure 1.

$$IE=E_{M{\left(H_{2}O\right)}_{n}}-\left(E_{M}+E_{{\left(H_{2}O\right)}_{n}}\right)$$ (1)

$$\Delta E_{\mathrm{seq}}=E_{M{\left(H_{2}O\right)}_{n}}-\left(E_{M{\left(H_{2}O\right)}_{n-1}}+E_{H_{2}O}\right)$$ (2)

where $E_{M{\left(H_{2}O\right)}_{n}}$ is the total energy of the metal ion–water complex, $E_{M}$ is the energy of the metal ion, $E_{{\left(H_{2}O\right)}_{n}}$ is the energy of water molecules, $E_{M{\left(H_{2}O\right)}_{n-1}}$ is the energy of the metal ion–water complex after taking away a water molecule, and $E_{H_{2}O}$ is the energy of a water molecule that has been taken away. The energies of the monomers, that is, $E_{M},E_{{\left(H_{2}O\right)}_{n}},E_{M{\left(H_{2}O\right)}_{n-1}}$ , and $E_{H_{2}O}$ , are computed on the frozen geometries as they occur in the complex with the metal ion. Natural population analysis (NPA) was performed to examine the charge transfer between the Sc ion and surrounding water molecules. All these calculations were performed using Gaussian16 software (Frisch et al., 2009).To obtain deeper insights into the intermolecular interactions, energy decomposition analysis was carried out using the LMOEDA scheme, implemented in the GAMESS program (Su et al., 2009). In the LMOEDA scheme, interaction energy (∆E_int_) decomposes into electrostatic (∆E_ele_), exchange (∆E_ex_), repulsion (∆E_rep_), polarization (∆E_pol_), and dispersion (∆E_disp_) components, as shown in Eq. 3:

$$\Delta {\text{E}}_{\text{int}}=\Delta {\text{E}}_{\text{ele}}+\Delta {\text{E}}_{\text{ex}}+\Delta {\text{E}}_{\text{rep}}+\Delta {\text{E}}_{\text{pol}}+\Delta {\text{E}}_{\text{disp}}$$ (3)

In LMOEDA, ∆E_ele_ describes the classical Coulomb interaction energy between the occupied orbitals of interacting moieties. The ∆E_pol_ component describes the orbital relaxation energy that comprises both polarization and charge-transfer interactions. The ∆E_ex_ and ∆E_rep_ components are associated with Pauli’s exclusion principle where ∆E_ex_ occurs in like-spin only, whereas ∆E_rep_ is expressed by the orthonormal orbitals of monomers. The ∆E_disp_ component alludes to the MP2 correction to the Hartree-Fock interaction energy. Bader’s theory of atoms in molecules (AIM) was used to analyze the topological parameters at critical points (CPs) for the considered systems using the AIM2000 package (Bader, 1985), and the relation of kinetic energy density, that is, G(**r**), and potential energy density, that is, V(**r**), was elucidated using Eqs. 4 and 5 to obtain the Laplacian of electron density (∇^2^ρ) and total energy density, that is, H(**r**)

$$\frac{1}{\;4}\nabla^{2}\rho =2G\left(r\right)+V\left(r\right)$$ (4)

H(r)=G(r)+V(r) (5)

## Results and Discussion

### Geometrical Parameters and Ground-State Multiplicity Prediction

**FIGURE 2:**
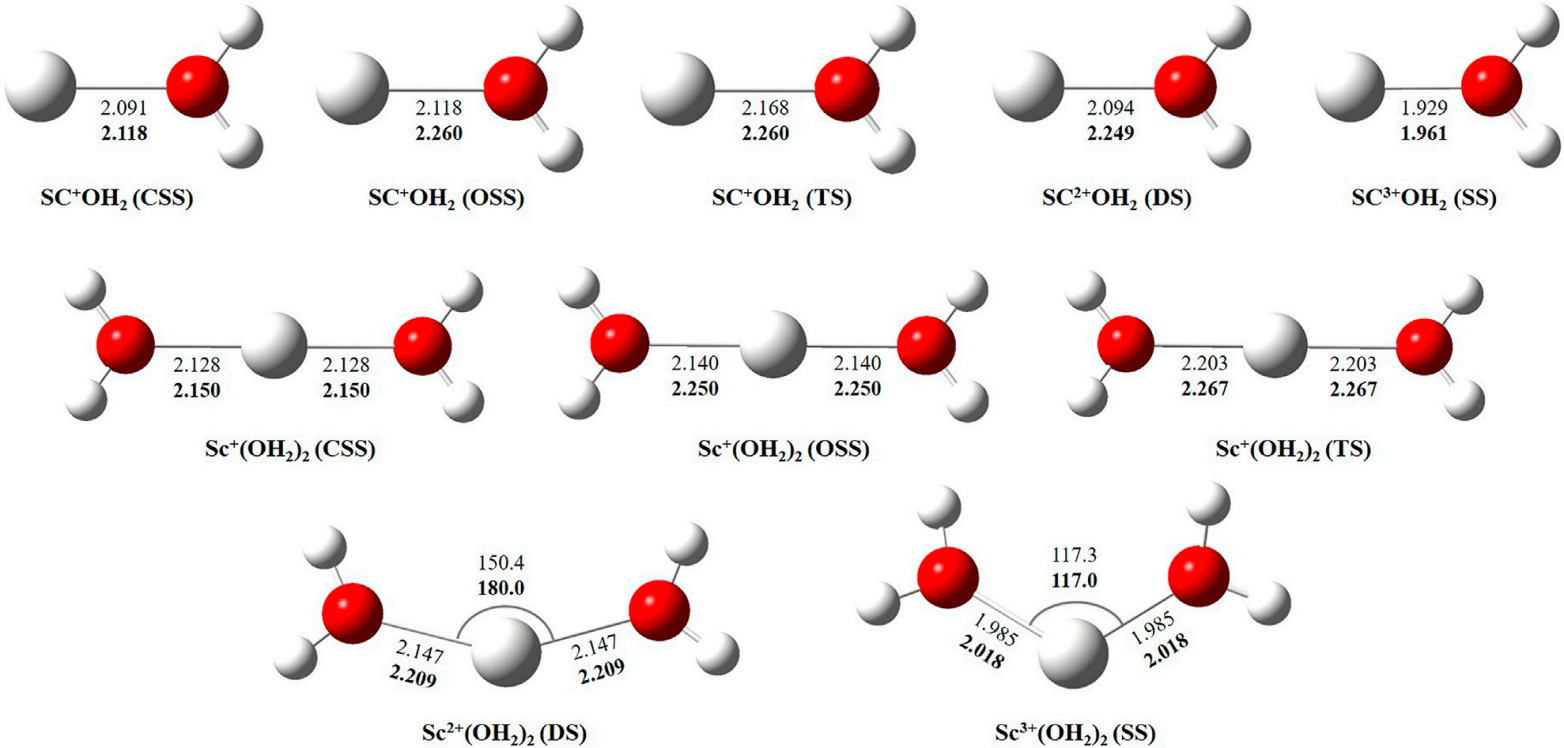
Geometrical parameters of the close-shell singlet (CSS), open-shell singlet (OSS), and triplet-state (TS) geometry of Sc^+^(OH_2_)_n_, doublet-state (DS) geometry of Sc^2+^(OH_2_)_n_, and singlet-state (SS) geometry of Sc^3+^(OH_2_)_n_, where n = 1-2, complexes obtained at B3LYP (normal) and MP2 (bold) using the 6-31G* basis set. Bond distances and angles are given in angstrom and degree, respectively.

**FIGURE 3:**
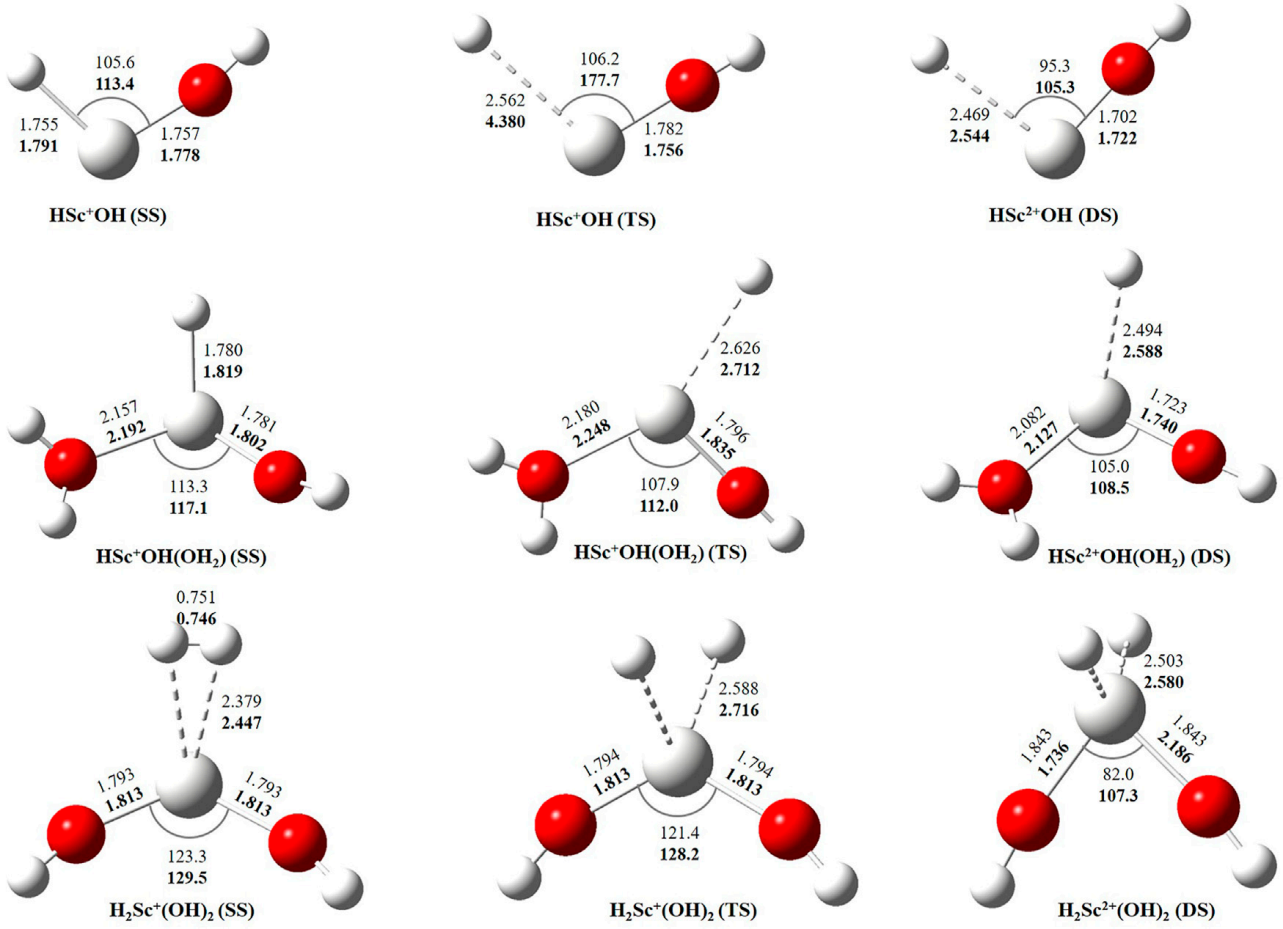
Geometrical parameters of singlet-state (SS), doublet-state (DS), and triplet-state (TS) geometries of HSc^+/2+^OH, HSc^+/2+^OH(OH_2_), and H_2_Sc^+/2+^(OH)_2_ complexes obtained at B3LYP (normal) and MP2 (bold) using the 6-31G* basis set. Bond distances and angles are given in angstrom and degree, respectively.

In this study, we have tried to adequately approximate the singlet–triplet energy gap (ΔS−ΔT), and therefore, open-shell singlet (OSS) and close-shell singlet (CSS) geometries of Sc^+^(OH_2_)_n_, where n = 1–2, complexes have also been considered. Geometrical parameters showed that OSS geometries of Sc^+^(OH_2_)_n_, where *n* = 1–2, complexes are closer to the triplet-state (TS) geometry. The geometrical parameters at B3LYP/6-31G* and MP2/6-31G* are shown in Figures 2, 3 and showed good agreement with results reported by Trachtman et al., 1998, Irigoras et al., 1999b, and Russo et al., 2001. The OSS geometry is obtained to be very similar to that of TS than the CSS because of its similarity in the orbital occupancy to the TS. Additionally, analysis of the geometrical parameters showed that the Sc^+^–O bond distance increases, and the H–O–H bond angle decreases as we move from low-spin complexes to high-spin complexes. The geometrical parameters of the water molecules slightly change while forming Sc^+^(OH_2_)_n_, where n = 1–2, complexes. For instance, the H–O–H bond angle of the water molecule changes around 3–4°during complexation, whereas an appreciable difference is not observed in the O–H bond distance of the water molecule. The O–Sc^+^–O bond angle of Sc^+^(OH_2_)_2_ complexes in both the singlet state (SS) and triplet state (TS) is observed to be 180.0° (linear molecular geometry); however, a nonlinear molecular geometry (O–Sc^+^–O bond angle of 117.3°) is observed for the tricationic complex at both B3LYP/6-31G* and MP2/6-31G* levels of theory. It can be noticed from Figure 2 that the MP2 method predicts a linear molecular geometry (180.0°) for the Sc^2+^(OH_2_)_2_ complex, whereas B3LYP predicts a nonlinear molecular geometry (150.4°). As a result, the O–Sc^2+^–O bond angle indicates structural relaxation upon the use of the MP2 method. In the case of insertion complexes, large differences are observed in the Sc^+^–H bond distance of SS and TS complexes, as shown in Figure 3. The Sc^+^–H bond distance for HSc^+^OH complexes obtained in the SS and TS is 1.755 Å and 2.563 Å at B3LYP and 1.791 Å and 4.370 Å at MP2, respectively. However, it is 1.780 Å and 2.626 Å at B3LYP and 1.819 Å and 2.712 Å at MP2, respectively, in the case of HSc^+^OH(OH_2_) complexes. In the case of the H_2_Sc^+^(OH)_2_ complex, long Sc^+^–H bonds, that is, 2.379 Å and 2.588 Å at B3LYP and 2.447 Å and 2.716 Å at MP2, are obtained for SS and TS complexes, respectively. Thus, the computed Sc^+^–H bond of the low-spin insertion complex was found to be substantially shorter compared to that of the high-spin complex. Hereby, long Sc^+^–H bond-containing insertion complexes may essentially correspond to H + Sc^+^OH, H + Sc^+^OH(OH_2_), H_2_ + Sc^+^(OH)_2_, H + Sc^2+^OH, H + Sc^2+^OH(OH_2_), H_2_ + Sc^+^(OH)_2_, and H + Sc^2+^OH rather than HSc^+^OH and HSc^2+^OH complexes. Therefore, the bond between the Sc ion and H of these insertion complexes is largely due to the electrostatic interaction, and also, the interaction is not entirely covalent in nature (Figure 3). A careful observation of the figure reveals that the trends of the structural parameters and their absolute values are very comparable, and thus, the qualitative results obtained in the study appear to be independent of the method employed. Therefore, to make a consistent choice of geometry, for further analysis, the structures optimized at the MP2/6-31G* level of theory have been undertaken.

The accurate estimation of the relative energy of spin states of transition metal ions is very important to describe the reactivity and other properties of these ions (Shaik et al., 2011; Costas et al., 2013). Since the energetics of transition metal ions are extremely difficult to predict accurately using any one computational method (Ashley et al., 2017), we have predicted the relative energy of SS and TS bare Sc^+^ ions, Sc^+^–OH_2_, and HSc^+^OH complexes at 48 different levels of theory. These methods are HF, PBEPBE, B3LYP, M06, MP2, and CCSD(T) using LanL2DZ, DGDZVP, Def2TZVP, 6-31G*, 6-311+G*, 6-311G**, cc-pVTZ, and aug-cc-pVTZ basis sets, as shown in Supplementary Table S1A. To calculate an adequate approximation of ΔS–ΔT for the bare Sc^+^ ion and complexes, the procedure proposed by Yamaguchi et al., 1988 is used to correct the spin contamination problem of the OSS bare Sc^+^ ion. The calculated ΔS–ΔT values of the bare Sc^+^ ion, Sc^+^OH_2_, and HSc^+^OH complexes are shown in Supplementary Tables S1, S2. A total of 14 different methods predicted a reasonably good value of ΔS−ΔT, that is, relative energy of contamination-corrected open-shell singlet (CC_OSS) and TS of the bare Sc^+^ ion, and showed an excellent agreement with the experimental value (7.38 kcal/mol) reported by Chen et al., 1994, as shown in Table 1. As expected, TS (3d^1^ 4s^1^) is predicted as a ground-state multiplicity for the bare Sc^+^ ion. These 14 methods, except M06, have also predicted TS (3d^1^ 4s^1^) as a ground-state multiplicity for the Sc^+^OH_2_ complex. In contrast, M06 predicted SS (4s^2^ 3d^0^) as a ground-state multiplicity for the Sc^+^OH_2_ complex, as shown in Supplementary Table S1B. The values obtained at PBEPBE (with few exceptions), B3LYP, MP2, and CCSD(T) showed a reasonable agreement with the reported theoretical values (8.63 kcal/mol to 20.30 kcal/mol) by Irigoras et al., 1999a. However, all the considered methods predicted SS (4s^2^ 3d^0^) as the ground-state multiplicity for insertion complexes.

**TABLE 1:** Reaction energy (ΔE_R_) for the reaction Sc^+^ + OH_2_ → Sc^+^O+ H_2_ + ΔE, sequential binding energy ( ${\text{ΔE}}_{\text{seq}}$ ) of ground-state Sc^+^OH_2_ complex, and ΔS−ΔT for the bare Sc^+^ ion. All values are reported in kcal/mol.

Methods	ΔS−ΔT	ΔE_seq_	ΔE_R_
HF/LanL2DZ	9.31	–41.08	–13.93
B3LYP/DGDZVP	7.80	–37.19	38.66
B3LYP/6-31G*	6.30	–41.49	53.79
B3LYP/aug-cc-pVTZ	8.00	–33.73	37.78
M06/Def2TZVP	8.62	–36.61	51.43
M06/6-31G*	5.25	–42.21	64.69
M06/6-311+G*	7.32	–40.08	58.36
M06/cc-pVTZ	8.54	–37.86	52.44
M06/aug-cc-pVTZ	8.88	–35.30	50.05
MP2/LanL2DZ	8.59	–42.37	66.30
MP2/6-311+G*	9.48	–34.98	53.61
MP2/cc-pVTZ	8.80	–31.94	46.16
MP2/aug-cc-pVTZ	8.75	–28.76	43.44
CCSD(T)/6-311G**	6.46	–31.12	35.88
Exp	7.38	–31.40 ± 3	46.81 ± 1.38

### Topological Analysis of Bonds

**FIGURE 4:**
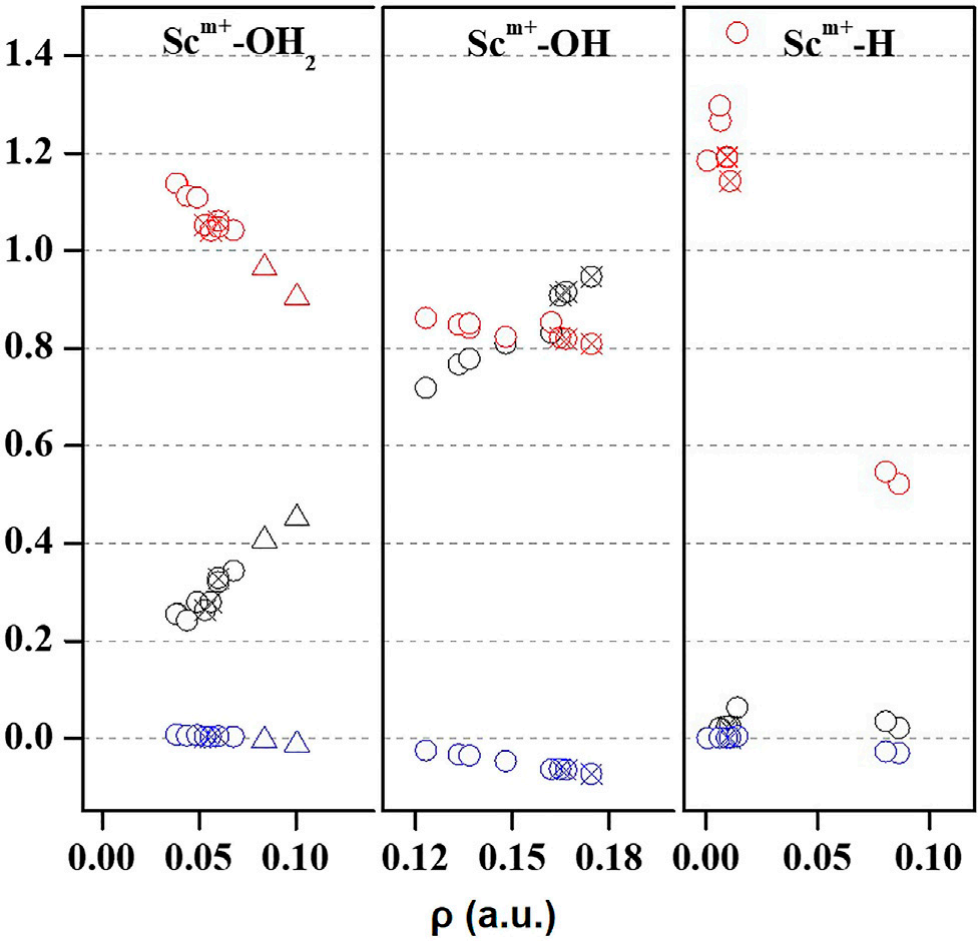
Variation of Laplacian of electron density (∇^2^ρ; black), total energy density (H(**r**); blue), and the ratio of kinetic energy density, that is, G(**r**), and potential energy density ([−G(**r**)/V(**r**)]; red) with respect to the electron density ( ρ ) obtained at BCP of Sc^+^–OH_2_, Sc^+^–OH, and Sc^+^–H bonds of mono- (circle), di- (cross circle), and tricationic (triangle) water and its insertion complexes.

Quantum theory of atoms in molecules (QTAIM) is useful to examine the interaction between atoms based on the topology of the electron density at CPs (Bader, 1998). We have examined ρ, ∇^2^ρ, H(**r**), [-(G(**r**)/V(**r**)], and percentage contribution of G(**r**) at the bond critical point (BCP) of the MP2/6-31G* optimized geometries of Sc ion–water and their insertion complexes to elucidate the nature of the Sc-ion bond with H_2_O, OH, and H, as shown in Figure 4 and Supplementary Table S3. A high value of ρ and a negative value of ∇^2^ρ indicate covalent interaction, while in general, a low value of ρ and a positive value of ∇^2^ρ suggest a noncovalent or close-shell type bonding (Kumar et al., 2021). However, the sign of H(**r**) and the value of [-G(**r**)/V(**r**)] explain the covalent nature of the bond. It may be noticed that the value of ρ increases and that of ∇^2^ρ and H(**r**) decreases for the Sc–O bond of Sc^+/2+/3+^–OH_2_ and Sc^+/2+^–OH complexes when moving from low-spin to high-spin complexes, corresponding to a covalent interaction. (Figure 4 **)**. However, in the case of Sc^+^(OH_2_)_n_, where n = 1–2, and HSc^+/2+^OH(OH_2_) complexes, a positive value of both ∇^2^ρ and H(**r**) with a small value of ρ (0.03–0.06 a.u.) and [−G(**r**)/V(**r**)] > 1 has been observed at the BCP of the Sc^+/2+^–OH_2_ bond, suggesting a noncovalent bond. A positive value of ∇^2^ρ and a negative value of H(**r**) with a significant value of ρ (∼0.10 a.u.) and 1 <[−G(**r**)/V(**r**)]> 0.5 suggest that the Sc^3+^–water bond possesses a partial covalent character. In the case of insertion complexes, a positive value of ∇^2^ρ and a negative H(**r**) with a ρ value of 0.08–0.17 a.u. have been observed for Sc^+^–OH, Sc^2+^–OH, and Sc^+^–H bonds, as shown in Figure 4 and Supplementary Table S3A. The value of [−G(**r**)/V(**r**)] is observed to be less than 1 (Figure 4), and the percentage contribution of G(**r**) into H(**r**) (Supplementary Table S3B) was less than 50% at the BCP of Sc^+/2+^–OH and Sc^+^–H bonds of HSc^+/2+^OH and HSc^+/2+^OH(OH_2_) complexes, that is, the lowering of the kinetic energy may be traced to the enhancement of the covalent nature in these bonds (Zhao et al., 2019). Besides, it may also be noted that the contribution of G(**r**) decreases with the increase in ρ at the BCP of the bond in most of the cases except for Sc^+/2+^–H bonds, which are clearly indicated as loose bonds. Furthermore, the bond length of Sc^+/2+^–H is long, clearly indicating that the Sc^+/2+^–H bond is not formed in the insertion complexes on the TS and doublet-state (DS) potential energy surfaces (see Supplementary Table S3A). Thus, topological analysis of Sc ion–water and its insertion complexes indicated that Sc^+/2+^–OH_2_ bonds are noncovalent in nature. A closer look at the structural and topological parameters reveals that Sc^3+^–OH_2_, Sc^2+^–OH, and Sc^+^–H bonds have a strong admixture of covalent nature.

### Analysis of Binding and Reaction Energies

The binding and reaction energies are calculated using the aforementioned 14 methods such as HF in conjugation with the LanL2DZ basis set, B3LYP in conjugation with DGDZVP, 6-31G*, and aug-cc-pVTZ basis sets, M06 in conjugation with 6-31G*, 6-311+G*, cc-pVTZ, and aug-cc-pVTZ basis sets, MP2 in conjugation with LanL2DZ, 6-311+G*, cc-pVTZ, and aug-cc-pVTZ basis sets, and CCSD(T) in conjugation with the 6-311G** basis set, as shown in Table 1 and Supplementary Table S4. The computed Sc^+^–OH_2_ binding energy is in fair agreement with the experimental observations and earlier computational results for the ground state of the Sc^+^OH_2_ complexes (Magnera et al., 1989) (Table 1). In comparison to the other theoretical values, our values calculated at B3LYP/aug-cc-pVTZ, MP2/cc-pVTZ, and CCSD(T)/6-311G** levels of theory showed a very good agreement with the value reported by Irigoras et al., 1999a and Sharma et al., 2016, that is, −32.52 kcal/mol and −31.18 kcal/mol at CCSD(T)/TZVP + G(3df2p) and CCSD(T)/def2-TZVP levels of theory, respectively. Moreover, our calculated values suggested that binding energy increases by moving from high spin to low spin and from lower to higher oxidation states, that is, from monocationic to tricationic, as shown in Supplementary Table S4. Figures 2 and 3 depict the optimized geometric parameters with two water molecules and provide interesting insights into the onset of microsolvation of the metal ion and the change in the propensity of competing pathways, in the presence of two water molecules. Rosi et al., 1989 have reported a similar study which is in good qualitative agreement with the results obtained here. Calculated values suggested that ΔE_seq_ decreases with the addition of the second water molecule to the Sc ion in all the considered methods (Supplementary Table S4). Reaction energy (ΔE_R_) has been calculated using the equation Sc^+^ + OH_2_→ Sc^+^O+ H_2_ + ΔE, as shown in Table 1. A large inconsistency is observed among the calculated ΔE values using 14 different methods. The results clearly reveal the grossly poor performance of the bulk of methods employed in estimating the reaction energies. The calculated values at B3LYP in conjugation with DGDZVP and aug-cc-pVTZ basis sets, MP2 in conjugation with cc-pVTZ and aug-cc-pVTZ basis sets, and CCSD(T) in conjugation with the 6-311G** basis set have shown good agreement with the experimental value (Chen et al., 1994). Thus, an exothermic reaction and the formation of the low-spin Sc^+^O+ H_2_ product are observed. In addition, our calculated values at MP2 in conjugation with cc-pVTZ and aug-cc-pVTZ, B3LYP in conjugation with DGDZVP and aug-cc-pVTZ, and CCSD(T) in conjugation with 6-311G** basis sets showed good agreement and seem to be better than other reported theoretical values (Tilson et al., 1991; Ye et al., 1997; Irigoras et al., 1999b). The binding and reaction energy values have also been computed at BP86, B97D, and B3LYP-D3 using 6-311G** and aug-cc-pVTZ basis sets, and the obtained values are observed to be similar to those observed using other DFT methods, as shown in Table 1 and Supplementary Table S10.

Thus, the calculated values ΔS−ΔT, ${\text{ΔE}}_{\text{seq}}$ , and ΔE_R_ suggested that B3LYP in conjugation with DGDZVP and aug-cc-pVTZ, MP2 in conjugation with 6–311+G*, cc-pVTZ, and aug-cc-pVTZ, and CCSD(T) in conjugation with the 6-311G** basis set predict the energetics of Sc ions accurately, and therefore, these methods can be used to delineate the interaction of the Sc ion with water molecules. We have opted the CCSD(T)/6-311G**//MP2/6-31G* method for further analysis.

### Potential Energy Surface Analysis

**FIGURE 5:**
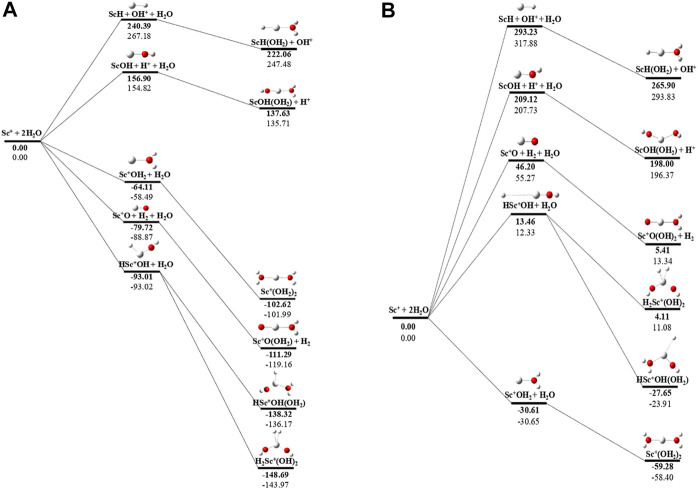
CCSD(T)/6-311G**//MP2/6-31G* (bold) and MP2/6-311G**//MP2/6-31G* (normal) energies (kcal/mol) for **(A)** singlet-state (SS) and **(B)** triplet-state (TS) potential energy surfaces following Sc^+^ + 2H_2_O reactions. The sum of reactant pair energy (Sc^+^ + 2H_2_O) is considered as a standard (energy zero) to calculate the relative energy.

**FIGURE 6:**
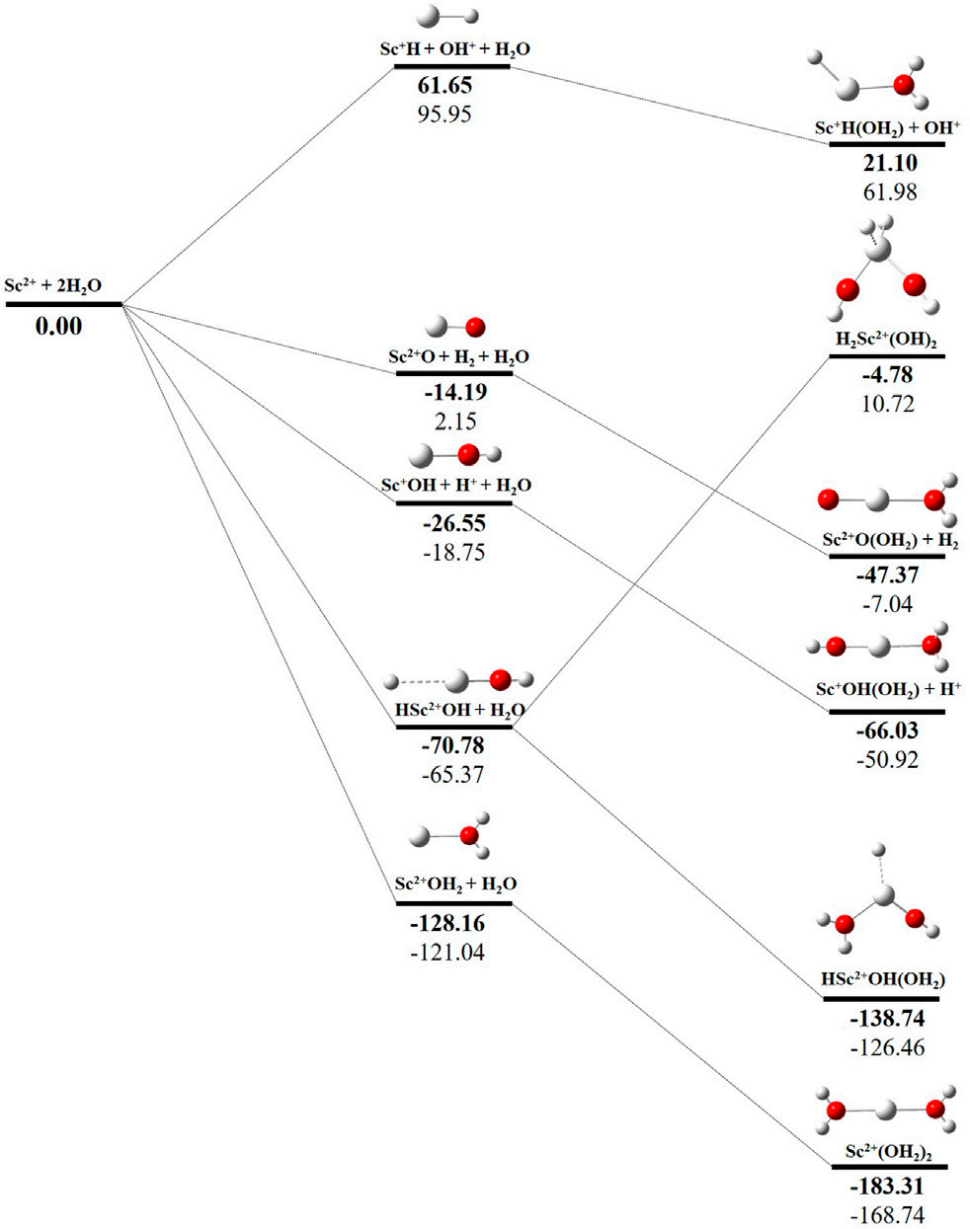
CCSD(T)/6-311G**//MP2/6-31G* (bold) and MP2/6-311G**//MP2/6-31G* (normal) energies (kcal/mol) for doublet-state (DS) potential energy surfaces following Sc^2+^ + 2H_2_O reaction. The sum of reactant pair energy (Sc^2+^ + 2H_2_O) is considered as a standard (energy zero) to calculate the relative energy.

**FIGURE 7:**
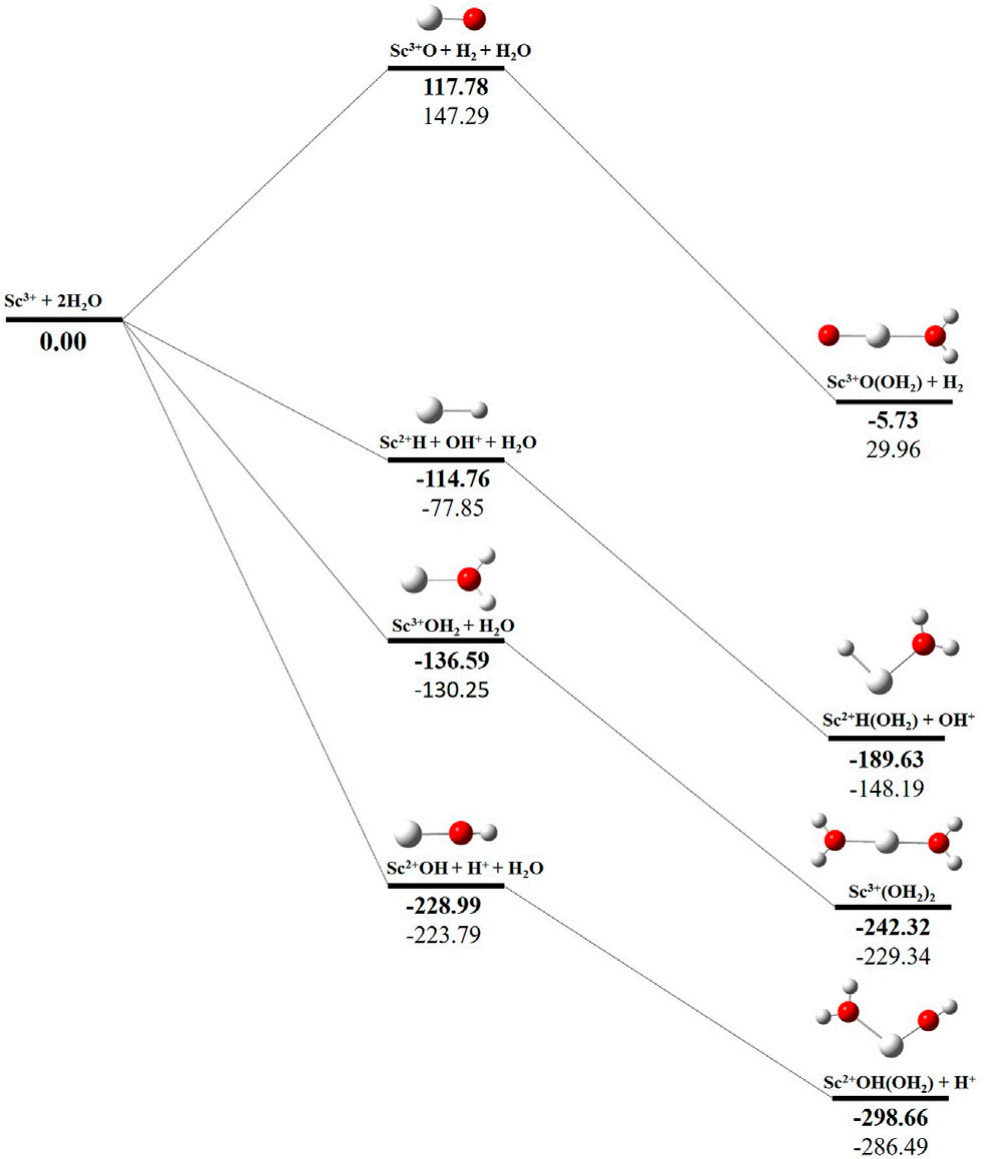
CCSD(T)/6-311G**//MP2/6-31G* (bold) and MP2/6-311G**//MP2/6-31G* (normal) energies (kcal/mol) for singlet-state (SS) potential energy surfaces following Sc^3+^ + 2H_2_O reaction. The sum of reactant pair energy (Sc^3+^ + 2H_2_O) is considered as a standard (energy zero) to calculate the relative energy.

The potential energy surface analysis has been carried out for interaction of the Sc^z+^ ion with water molecules leading to 1) hydrate: Sc^z+^(H_2_O)_n_, 2) hydrolyse: H–Sc^z+^–OH (H_2_O)_n-1_orH_2_–Sc^z+^–(OH)_2_(H_2_O)_n-2_, 3) dehydrogenate: Sc ^z+^O+ H_2_, 4) metal hydroxide: Sc^(z−1)+^OH, and 5) metal hydride: Sc^(z−1)+^H at CCSD(T)/6-311G**//MP2/6-31G** level of theory, as shown in Figures 5–7). No stationary point on the potential energy surface has been obtained corresponding to the OSS complex of Sc^+^OH_2_, as the putative structure collapses to CSS in a barrierless fashion upon the formation of the insertion complex (HSc^+^OH), which corroborates well with the earlier observations (Irigoras et al., 1999a). The reaction starts from bare Sc^+^ ions separated from water molecules at an infinite distance and leads to the formation of the hydrated complex, insertion complex, metal oxide, metal hydroxide, and metal hydride, as shown in Supplementary Figure S1. Considering the well-known limitations of the MP2 methods, when dynamic electron correlation is substantial, in properly reproducing the geometry of small systems, we would like to benchmark the method more systematically. Thus, geometry optimization of the considered metal oxide, hydroxide, and hydride complexes has been performed at five different levels of theory. The insertion complex formed by Sc^+^ insertion into the O–H bond of water molecules, as the valance electrons of Sc^+^ ions are shared by -OH and -H atoms, making the complex a singlet, Figures 5–7, which depicts the energetics of the competing pathways, from which it can be seen that the insertion complex, that is, HSc^+^OH, is substantially stable in the singlet compared to that of the triplet, while the energetics of the spin states of the bare metal ion exhibit completely contrasting trends. The observed spin-crossover following the insertion reveals that there is a possibility for multiple electronic states crossing along the reaction paths on the potential energy surfaces. Thus, modeling of these molecules warrants careful consideration of various electronic states as there is a great potential for crossover of the spin and electronic states due to the vast variation in the energetics of these species even with small geometric changes along with the reaction coordinate. The insertion complex, on the triplet potential energy surface, is essentially a hydrogen elimination process with the formation of a low-spin Sc^+^O+ H_2_ as the product, which is in agreement with the earlier study by Irigoras et al., 1999b. Thus, the metal ions under the right condition have a definitive potential to generate hydrogen, which is of course of great industrial significance. In the case of metal oxide, a relatively stable Sc^+^O complex has been observed on the SS potential energy surface than the corresponding Sc^+^OH_2_ complex. However, HSc^+^OH is relatively more stable than the Sc^+^O complex on the SS potential energy surface. The energetics of the formation of a metal hydroxide ScOH and metal hydride ScH suggest that both the reactions are energetically unfavorable on both SS and TS potential energy surfaces. We have further expanded the scope of this study by examining the interaction of monohydrated, insertion, oxide, hydroxide, and hydride complexes with the second water molecule. The hydrated products of monohydrated, insertion, oxide, hydroxide, and hydride complexes are observed to be more stable than their predecessors on both SS and TS potential energy surfaces, as shown in Figure 5. Interestingly, the hydrated TS insertion complex H^**…**^Sc^+^OH(OH_2_) is lower than both TS insertion complexes, H^…^Sc^+^OH and the reactant pair Sc^+^ + 2H_2_O by 41.11 kcal/mol and 27.65 kcal/mol, respectively, and nearly degenerates (∼2.96 kcal/mol) to the TS monohydrated complex Sc^+^OH_2_. It can be inferred that splitting the second water molecule slightly destabilizes the TS monohydrated complex, and thus, two water molecules appeared to be required to reach a stable complex on the potential energy surface. Thus, when performing the computations with two water molecules, Sc^+^ insertion into the O–H bond of the second water molecule eventually forms metal dihydroxide, that is, Sc^+^(OH)_2_ + 2H on the TS potential energy surface and Sc^+^(OH)_2_ + H_2_on the SS potential energy surface. It is interesting to observe that there is a great propensity exhibited for water splitting compared to solvation, with the Sc^+^(OH)_2_ + H_2_ product on the SS being observed well below the ground-state reactant pair Sc^+^ + 2H_2_O and Sc^+^(OH_2_)_2_ complexes. The examination of di- and tricationic Sc-ion reactions with a water molecule indicates that the hydrated complex of Sc^2+^ and hydroxide of Sc^3+^ lie below the reactant and the corresponding complexes on the DS and SS potential energy surfaces, respectively (Figures 6, 7). Therefore, it may be inferred that the Sc^2+^ ion is likely to adopt a more feasible solvation pathway compared to the corresponding insertion alternative (Figure 6). However, the affinity of the Sc^3+^ ion to form a metal hydroxide, Sc^2+^OH, is observed to be higher than getting solvated to form Sc^3+^(OH_2_) as hydroxide complexes lie above the corresponding hydrated complex (Figure 7).

### Analysis of Sequential Solvation

**FIGURE 8:**
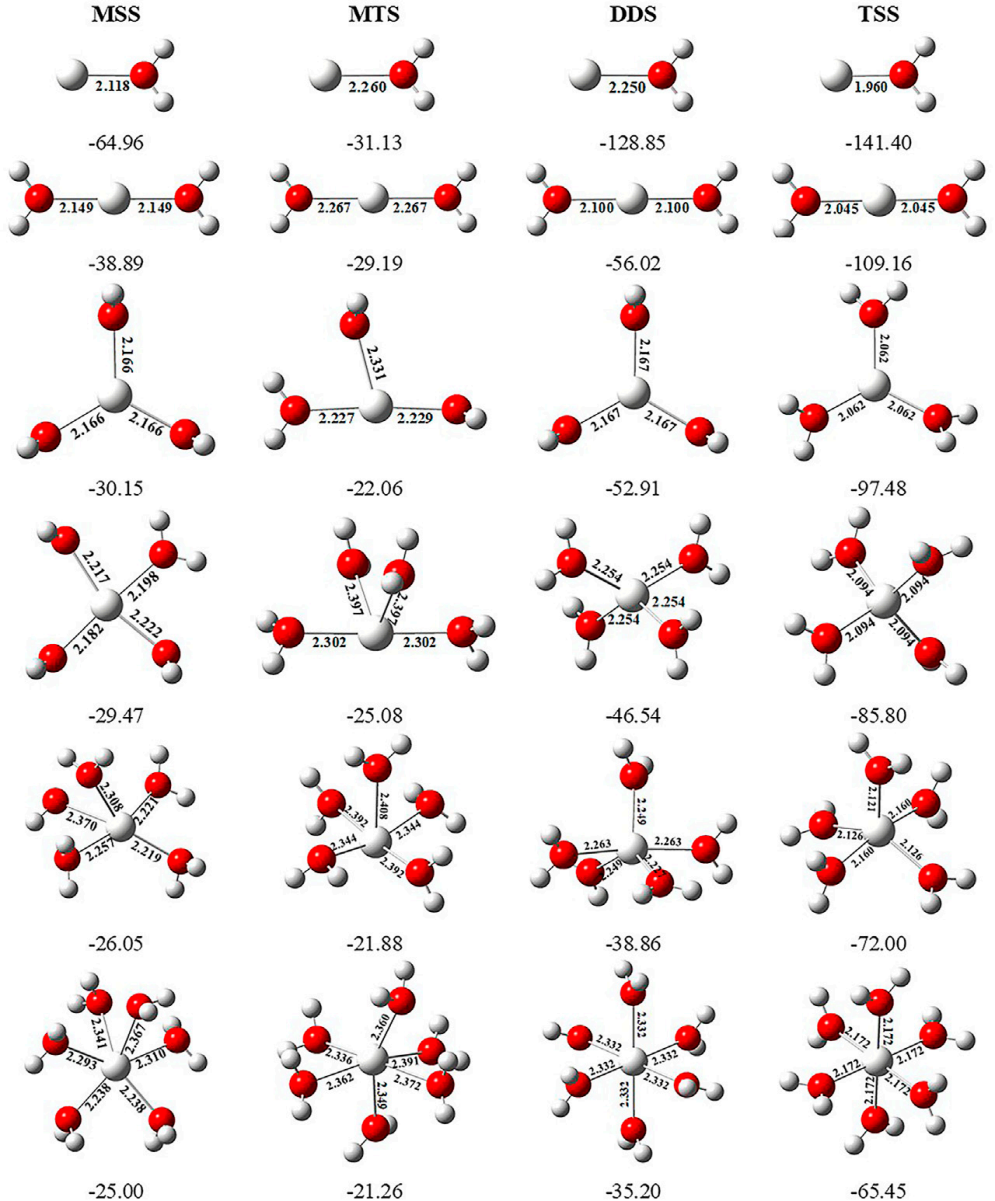
Geometrical parameters (bond distances in angstrom) of the monocationic singlet state (MSS) and triplet state (MTS), dicationic doublet state (DDS), and tricationic singlet state (TSS) of Sc^z+^(OH_2_)_n_, where z = 1–3 and n = 1–6, ion complexes, obtained at the MP2/6-31G* level of theory. Sequential binding energy (ΔE_seq_; kcal/mol) of these complexes obtained at the CCSD(T)/6-311G**//MP2/6-31G* level of theory.

**FIGURE 9:**
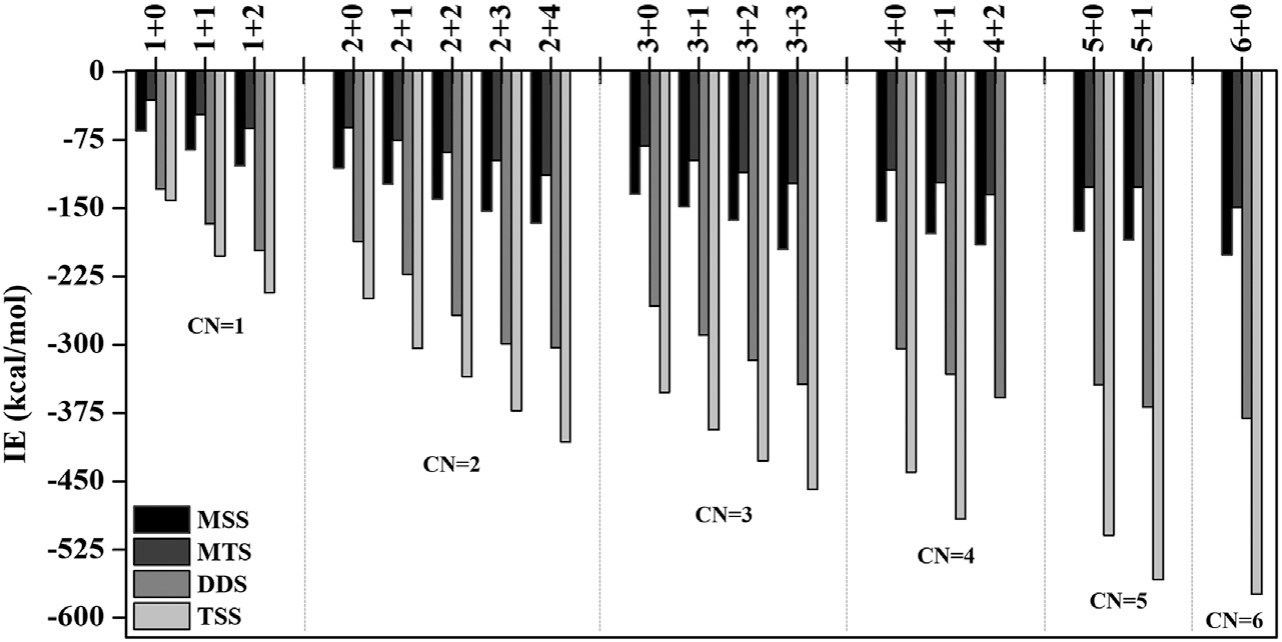
Variation of interaction energy (IE in kcal/mol) with the increase in coordination number (CN) of the monocationic singlet state (MSS) and triplet state (MTS), dicationic doublet state (DDS), and tricationic singlet state (TSS) of the Sc ion in Sc^z+^(OH_2_)_n_; *z* = 1–3 and *n* = 1–6 complexes calculated at the CCSD(T)/6-311G**//MP2/6-31G* level of theory.

In order to investigate the sequential solvation effect on the nature of Sc ion–water complexes, we have examined the microsolvation process of up to 6 water molecules and probed the relative propensity of water molecules to occupy the first and second solvation shells. The schematic depiction of the representative structure of Sc(OH_2_)_n_, where n = 1–6, ion complexes and their nomenclature are shown in Figure 1. We have used the nomenclature X + Y (X = number of water molecules in the first solvation shell and Y = number of water molecules in the second solvation shell) to present the number of water molecules in the solvation shells. Starting from one water molecule, various conformers of Sc(OH_2_)_n_, where n = 1–6, ion complexes in all possible oxidation and spin states have been explored and the lowest energy conformers are selected for further discussion, as shown in Figure 8 and Supplementary Figure S3. A cursory look of interaction energy (IE) of these complexes, obtained at the CCSD(T)/6-311G**//MP2/6-31G** level of theory, suggests that IE increases as the number of water molecules increase either in the first or both in the first and second solvation shells, as shown in Figure 9 and Supplementary Figure S2. IE has also been obtained using different methods to ensure that the obtained values are not biased (Supplementary Table S5).We have considered IE of each structure as a criterion to describe the stability of Sc(OH_2_)_n_, where n = 1–6, ion complexes. The sequential binding ΔE_seq_ energy of these stable complexes, obtained at the CCSD(T)/6-311G**//MP2/6-31G** level of theory, suggests that ΔE_seq_ energy decreases with the addition of every subsequent water molecule to the complexes except for the 4 + 0 structure of the triplet Sc^+^(OH_2_)_4_ complex, as shown in Figure 8. Our calculated values showed a good agreement with the available experimental and theoretical results reported by Rosi et al., 1989, Magnera et al., 1989, and Dalleska et al., 1994.

IE increases as the number of water molecules increases in the second solvation shell for each CN of Sc ions irrespective of oxidation and spin states, that is, it increases from 1 + 0 to 1 + 2 (CN = 1), 2 + 0 to 2 + 4 (CN = 2), 3 + 0 to 3 + 3 (CN = 3), 4 + 0 to 4 + 2 (CN = 4), and 5 + 0 to 5 + 1 (CN = 5), shown in Figure 9 and Supplementary Figure S2. Thus, this result suggests that Sc(OH_2_)_n_, where n = 1–6, ion complexes in each CN are more stable with the maximum number of water molecules in the second solvation shell. Furthermore, the analysis of the bond distance between the Sc ion and first solvation shell water molecules suggested that the average M–O distance of Sc(OH_2_)_n_, where n = 1–6, ion complexes increases with increasing number of water molecules around the Sc ion. Among the complexes with the same number of water molecules in the second solvation shell, the average M–O distance increases with the increase in the CN of the Sc ion and vice versa. In addition, it has also been observed that the average M–O distance increased by moving from low- to high-spin Sc^+^(OH_2_)_n_, where n = 1–6, ion complexes and decreased by moving from mono- to tricationic ground-state complexes, as shown in Supplementary Table S6. The topological analysis predicted Sc^+/2+^–OH_2_ bonds to be a noncovalent bond as the calculated values of ∇^2^ρ and H(**r**) are positive, and [−(G(**r**)/V(**r**)] is less than 1 irrespective of the spin states. However, the Sc^3+^–OH_2_ bond is predicted as partially covalent in nature, as shown in Supplementary Table S7.

### Charge and Energy Decomposition Analysis

**FIGURE 10:**
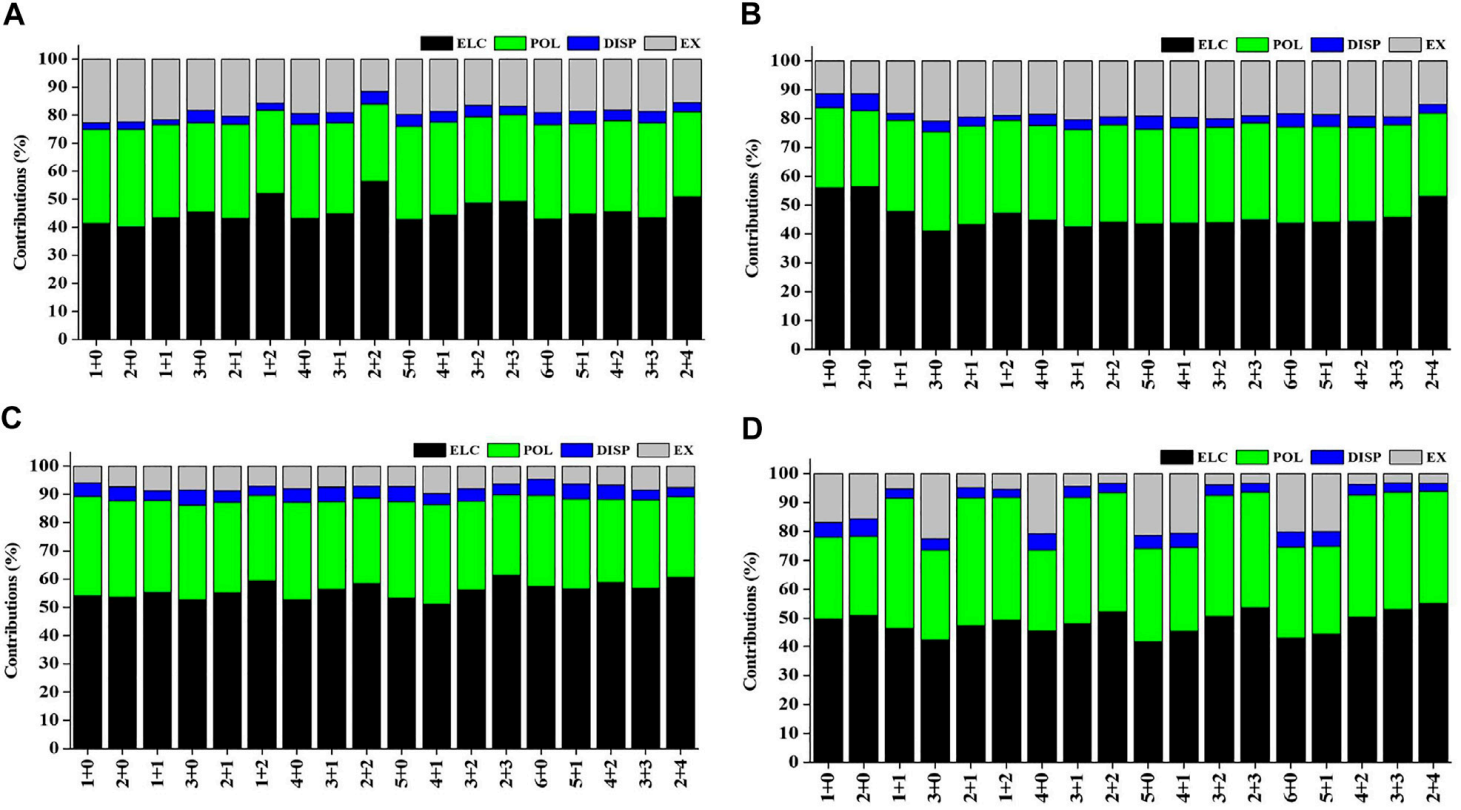
Percentage contribution of energy components such as electrostatic (ELC), polarization (POL), dispersion (DISP), and exchange (EX) into the interaction energy of the **(A)** monocationic singlet state (MSS), **(B)** monocationic triplet state (MTS), **(C)** dicationic doublet state (DDS), and **(D)** tricationic singlet state (TSS) of Sc^z+^(OH_2_)_n_; *z* = 1–3 and *n* = 1–6 complexes obtained using the LMO-EDA scheme at B3LYP/6-311G**//MP2/6-31G* for hydrogen (H) and oxygen (O) and B3LYP/cc-pVTZ//MP2/6-31G* for the Sc^z+^ ion.

Natural population analysis has been carried out on all the considered structures of Sc(OH_2_)_n_, where n = 1–6, ion complexes to examine the charge transfer between the Sc ion and the water molecules. The variation in charge on the Sc ion with the increase in the CN of Sc^+^(OH_2_)_n_, where n = 1–6, complexes is shown in Supplementary Figure S4. The figure shows that the charge on the Sc ion decreases monotonically as the number of water molecules is increased in the complexes. Besides, the charge on the Sc ion seemed to be decreasing as the number of water molecules increases in the second solvation shell for each coordination number (i.e., CN = 1–6) and vice versa. Such differences can be explained based on the differences in the organization of water molecules around the Sc ion. The most stable structure of every CN complex showed a minimum charge on the Sc ion. A cursory view of Supplementary Table S8 shows that IE is inversely proportional to the charge on the Sc ion, which signifies that charge transfer plays an important role in the stabilization of Sc(OH_2_)_n_, where n = 1–6, ion complexes. Localized molecular orbital energy decomposition analysis is carried out to examine the contribution of energy components into the Sc ion–water interaction, as shown in Figure 10 and Supplementary Table S9. The figure shows that the contribution of the ∆E_ele_ component dominates the Sc and water interaction followed by the contribution of the ∆E_pol_ component, irrespective of oxidation and spin states. However, the contribution of the ∆E_disp_ component is found to be negligible with around a contribution of 2–6%, as shown in Figure 10.

## Conclusion

The present study reveals that a bare metal ion, in general, and Sc ion, in particular, have a great propensity in participating in multiple mechanistic pathways, and the relative propensity and feasibility of these pathways are dependent on a number of factors. As expected, water molecules in the first solvation shell bestow higher levels of stability for the complexes compared to the situation where they are occupying the second solvation shell. Following similar trends, within the case for each coordination number (CN = 1–5), the complexes with the proportionately higher number of water molecules in the closer solvation shell are found to be more stable. The sequential solvation analysis suggested that Sc(OH_2_)_n_, where n = 1–6, ion complexes are stable when all water molecules are in the first solvation shell, that is, 1 + 0, 2 + 0, 3 + 0, 4 + 0, 5 + 0, and 6 + 0 structures. ${\text{ΔE}}_{\text{seq}}$ decreases with the addition of every subsequent water molecule to the complexes irrespective of oxidation and spin states, without any exception. The oxidation and spin states and the microenvironment around the ion–water interaction will have a profound influence on the relative feasibility of these competing pathways, giving rise to the power of fine-tuning the electrons and microenvironment around the metal to achieve the preference for a given pathway. Thus, transition metal catalysis to split water and produce important gases such as H_2_ and O_2_ is a very promising and viable approach, and the key is to electronically fine-tune the metal ion center.

## Data Availability

The raw data supporting the conclusion of this article will be made available by the authors, without undue reservation.
