# Supplementary material for: Fate of Sc-Ion Interaction With Water: A Computational Study to Address Splitting Water Versus Solvating Sc Ion
Source: Front Chem. 2021 Oct 18;9:738852. doi: 10.3389/fchem.2021.738852 (PMC8558820; doi:10.3389/fchem.2021.738852)
Supplement: Supplementary file 1 [file DataSheet1.docx]

Supplementary Material

**Fate of Sc Ion Interaction with Water: A Computational Study to Address Splitting Water Versus Solvating ScIon**

**Nandan Kumar^1,2^, Y. Bhargav Kumar^2,3^, Himakshi Sarma^3^, G. Narahari Sastry^1,2,3^***

^1^Centre for Molecular Modelling, CSIR-Indian Institute of Chemical Technology, Tarnaka, Hyderabad 500007, Telangana State, India.

^2^Academy of Scientific and Innovative Research (AcSIR), Ghaziabad, 201002, India.

^3^Advanced Computation and Data Sciences Division, CSIR-North East Institute of Science and Technology, Jorhat, 785006, Assam, India.

*** Correspondence:**Corresponding Author

[gnsastry@gmail.com](mailto:gnsastry@gmail.com); [gnsastry@neist.res.in](mailto:gnsastry@neist.res.in)

**Keywords**: Metal cation-water interaction; binding energy, electron density; Laplacian of electron density; energy decomposition analysis

**Supplementary Figures and Tables**

**Supplementary Figure 1.** Geometrical parameters of mono-cationic singlet state (MSS), mono-cationic triplet state (MTS), di-cationic doublet state (DDS), and tri-cationic singlet state (TSS) of ScH, ScO, and ScOH complexes optimized at MP2/6-31G* (bold), B3LYP/6-31G* (underline), MP2/6-311G** (underline italic), MP2/6-311++G** (italic), and B3LYP/6-311++G** (normal) levels of theory. Bond distance is given in angstrom.

**Supplementary Figure 2.** Interaction energy (IE in kcal/mol) of mono-cationic singlet state (MSS), and triplet state (MTS), di-cationic doublet state (DDS), and tri-cationic singlet state (TSS) of Sc^z+^(OH_2_)_n_; z = 1-3 and n = 1-6 complexes obtained at CCSD(T)/6-311G**//MP2/6-31G* level of theory.

**Supplementary Figure 3.**Geometrical parameters of mono-cationic singlet state (MSS), and triplet state (MTS), di-cationic doublet state (DDS), and tri-cationic singlet state (TSS) of Sc^z+^(OH_2_)_n_; z = 1-3 and n = 1-6 complexes, obtained at MP2/6-31G* level of theory. Bond distance is given in angstrom.

**Supplementary Figure 4.** Variation in charge on mono-cationic singlet state (MSS), and triplet state (MTS), di-cationic doublet state (DDS), and tri-cationic singlet state (TSS) of Sc ion with the increase in size of Sc^z+^(OH_2_)_n_; z = 1-3 and n = 1-6 complexes calculatedat CCSD(T)/6-311G**//MP2/6-31G* level of theory.

**Supplementary Table 1(A).** Relative energies (kcal/mol) calculated for open shell singlet (OSS), contamination corrected open shell singlet (CC_OSS), and close shell singlet (CSS) of bare Sc^+^ ion. Triplet state (TS) of bare Sc^+^ ion is considered as a standard (energy zero) to calculate the relative energy.

**Supplementary Table 1(B).** Relative energies (kcal/mol) calculated for singlet state (SS) of Sc^+^OH_2_ and HSc^+^OH complexes. Triplet state (TS) of Sc^+^OH_2_ and HSc^+^OH complexes is considered as a standard (energy zero) to calculate the relative energy.

**Supplementary Table 2.** List of the selected methods based on the calculated relative energies (kcal/mol) for open shell singlet (OSS), contamination corrected open shell singlet (CC_OSS), and close shell singlet (CSS) of bare Sc^+^ ion. Triplet state (TS) of bare Sc^+^ ion is considered as a standard (energy zero) to calculate the relative energy.

**Supplementary Table 3(A).** The variation of electron density ($\rho, in a.u.$), Laplacian of electron density ($\nabla^{2}\rho, in a.u.$), total energy density (H(**r**), $in a.u.$) and ration of potential and kinetic energy density ([-G(**r**)/V(**r**)]) as obtained at bond critical point of Sc-OH_2_, Sc-OH and Sc-H bonds of (ScOH_2_)_n_;n=1-2 ion- and their insertion complexes,obtained at MP2/6-31G* optimized geometry.

**Supplementary Table 3(B).** The absolute value of kinetic energy density (G(**r**)$in a.u.$), potential energy density (V(**r**)$in a.u.$), and the percentage contribution of G(**r**) into total energy density (H(**r**)) as obtained at bond critical point of Sc-OH_2_, Sc-OH and Sc-H bonds of (ScOH_2_)_n_;n=1-2 ion- and their insertion complexes,obtained at MP2/6-31G* level of theory.

**Supplementary Table 4.**Sequential binding energy (${\Delta E}_{\mathrm{seq}}$; kcal/mol) of mono-cationic singlet state (MSS), and triplet state (MTS), di-cationic doublet state (DDS), and tri-cationic singlet state (TSS) of Sc ion in Sc^z+^(OH_2_)_n_; z = 1-3 and n = 1-2 complexes using MP2/6-31G* optimized geometry.

**Supplementary Table 5(A).** Interaction energy (IE; kcal/mol) and nuclear repulsion energy (Hartree) of singlet Sc^+^(OH_2_)_n_; n=1-6 complexes obtained at CCSD(T)/6-311G**//MP2/6-31G* level of theory.

**Supplementary Table 5(B).** Interaction energy (IE; kcal/mol) and nuclear repulsion energy (Hartree) of triplet Sc^+^(OH_2_)_n_; n=1-6 complexes obtained at CCSD(T)/6-311G**// MP2/6-31G* level of theory.

**Supplementary Table 5(C).** Interaction energy (IE; kcal/mol) and nuclear repulsion energy (Hartree) of doublet Sc^2+^(OH_2_)_n_; n=1-6 complexes obtained at CCSD(T)/6-311G**//MP2/6-31G* level of theory.

**Supplementary Table 5(D).** Interaction energy (IE; kcal/mol) and nuclear repulsion energy (Hartree) of singlet Sc^3+^(OH_2_)_n_; n=1-6 complexes obtained at CCSD(T)/6-311G**// MP2/6-31G* level of theory.

**Supplementary Table 6.**Variation in average M-O distance (Å) of mono-cationic singlet state (MSS), and triplet state (MTS), di-cationic doublet state (DDS), and tri-cationic singlet state (TSS) of Sc(OH_2_)_n_; n=1-6 complexes, obtained at MP2/6-31G* level of theory.

**Supplementary Table 7(A).** The variation of electron density (ρ in a.u.), Laplacian of electron density (∇^2^ρ in a.u.), total energy density (H(**r**) in a.u.) and ration of kinetic and potential energy density ([-G(r)/V(r)]), obtained at bond critical point of Sc^+^-OH_2_ bonds of singlet Sc^+^(OH_2_)_n_; n=1-6 complexes, calculated at MP2/6-31G* level of theory.

**Supplementary Table 7(B).**The variation of electron density (ρ in a.u.), Laplacian of electron density (∇^2^ρ in a.u.), total energy density (H(**r**) in a.u.) and ration of kinetic and potential energy density ([-G(r)/V(r)]), obtained at bond critical point of Sc^+^-OH_2_ bonds of triplet Sc^+^(OH_2_)_n_; n=1-6 complexes, calculated at MP2/6-31G* level of theory.

**Supplementary Table 7(C).**The variation of electron density (ρ in a.u.), Laplacian of electron density (∇^2^ρ in a.u.), total energy density (H(**r**) in a.u.) and ration of kinetic and potential energy density ([-G(r)/V(r)]), obtained at bond critical point of Sc^2+^-OH_2_ bonds of doublet Sc^2+^(OH_2_)_n_; n=1-6 complexes, calculated at MP2/6-31G* level of theory.

**Supplementary Table 7(D).**The variation of electron density (ρ in a.u.), Laplacian of electron density (∇^2^ρ in a.u.), total energy density (H(**r**) in a.u.) and ration of kinetic and potential energy density ([-G(r)/V(r)]), obtained at bond critical point of Sc^3+^-OH_2_ bonds of singlet Sc^3+^(OH_2_)_n_; n=1-6 complexes, calculated at MP2/6-31G* level of theory.

**Supplementary Table 8(A).**NPA charge on Sc ion and interaction energy (IE in kcal/mol) of mono-cationic singlet state (MSS), and triplet state (MTS), di-cationic doublet state (DDS), and tri-cationic singlet state (TSS) of Sc^z+^(OH_2_)_n_; z = 1-3 and n = 1-6 complexes, obtained at CCSD(T)/6-311G**//MP2/6-31G* level of theory.

**Supplementary Table 8(B).**Spin density on Sc ion of mono-cationic triplet state (MTS), and di-cationic doublet state (DDS) of Sc^z+^(OH_2_)_n_; z = 1-3 and n = 1-6 complexes, obtained at CCSD(T)/6-311G**//MP2/6-31G* level of theory.

**Supplementary Table 9(A).** Contribution of energy components (kcal/mol) such as electrostatic (ELC), exchange (EX), repulsion (REP), polarization (POL), dispersion (DISP), and into the interaction energy of ground state Sc^+^(OH_2_)_n_; n=1-6 complexes obtained using LMO-EDA scheme at B3LYP/6-311G**//MP2/6-31G* for Hydrogen (H) and Oxygen (O) and B3LYP/cc-pVTZ//MP2/6-31G* for metal ion.

**Supplementary Table 9(B).** Contribution of energy components (kcal/mol) such as electrostatic (ELC), exchange (EX), repulsion (REP), polarization (POL), dispersion (DISP), and into the interaction energy of triplet Sc^+^(OH_2_)_n_; n=1-6 complexes obtained using LMO-EDA scheme at B3LYP/6-311G**//MP2/6-31G* for Hydrogen (H) and Oxygen (O) and B3LYP/cc-pVTZ//MP2/6-31G* for metal ion.

**Supplementary Table 9(C).** Contribution of energy components (kcal/mol) such as electrostatic (ELC), exchange (EX), repulsion (REP), polarization (POL), dispersion (DISP), and into the interaction energy of doublet Sc^2+^(OH_2_)_n_; n=1-6 complexes obtained using LMO-EDA scheme at B3LYP/6-311G**//MP2/6-31G* for Hydrogen (H) and Oxygen (O) and B3LYP/cc-pVTZ//MP2/6-31G* for metal ion.

**Supplementary Table 9(D).** Contribution of energy components (kcal/mol) such as electrostatic (ELC), exchange (EX), repulsion (REP), polarization (POL), dispersion (DISP), and into the interaction energy of singlet Sc^3+^(OH_2_)_n_; n=1-6 complexes obtained using LMO-EDA scheme at B3LYP/6-311G**//MP2/6-31G* for Hydrogen (H) and Oxygen (O) and B3LYP/cc-pVTZ//MP2/6-31G* for metal ion.

**Supplementary Table 10.** Reaction energy (ΔE_R_) for the reaction Sc^+^ + OH_2_🡪ScO^+^ + H_2_ + ΔE, and sequential binding energy (${\Delta E}_{\mathrm{seq}}$) of ground state Sc^+^OH_2_ complex. All values are reported in kcal/mol.

| **A** |
| --- |
|  |
| **B** |
|  |

**Supplementary Figure 1.** Geometrical parameters of mono-cationic singlet state (MSS), mono-cationic triplet state (MTS), di-cationic doublet state (DDS), and tri-cationic singlet state (TSS) of ScH, ScO, and ScOH complexes optimized at MP2/6-31G* (bold), B3LYP/6-31G* (underline), MP2/6-311G** (underline italic), MP2/6-311++G** (italic), and B3LYP/6-311++G** (normal) levels of theory. Bond distance is given in angstrom.

**Supplementary Figure 2.** Interaction energy (IE in kcal/mol) of mono-cationic singlet state (MSS), and triplet state (MTS), di-cationic doublet state (DDS), and tri-cationic singlet state (TSS) of Sc^z+^(OH_2_)_n_; z = 1-3 and n = 1-6 complexes obtained at CCSD(T)/6-311G**//MP2/6-31G* level of theory.

| **MSS** | **MTS** | **DDS** | **TSS** |
| --- | --- | --- | --- |
|  |  |  |  |
|  |  |  |  |
|  |  |  |  |
|  |  |  |  |
|  |  |  |  |
|  |  |  |  |
|  |  |  |  |
|  |  |  |  |
|  |  |  |  |
|  |  |  |  |
|  |  |  |  |
|  |  |  |  |

**Supplementary Figure 3.**Geometrical parameters of mono-cationic singlet state (MSS), and triplet state (MTS), di-cationic doublet state (DDS), and tri-cationic singlet state (TSS) of Sc^z+^(OH_2_)_n_; z = 1-3 and n = 1-6 complexes,obtained at MP2/6-31G* level of theory. Bond distance is given in angstrom.

**Supplementary Figure 4.** Variation in charge on mono-cationic singlet state (MSS), and triplet state (MTS), di-cationic doublet state (DDS), and tri-cationic singlet state (TSS) of Sc ion with the increase in size of Sc^z+^(OH_2_)_n_; z = 1-3 and n = 1-6 complexes calculatedat CCSD(T)/6-311G**//MP2/6-31G* level of theory.

**Supplementary Table 1(A).**Relative energies (kcal/mol) calculated for open shell singlet (OSS), contamination corrected open shell singlet (CC_OSS), and close shell singlet (CSS) of bare Sc^+^ ion. Triplet state (TS) of bare Sc^+^ ion is considered as a standard (energy zero) to calculate the relative energy.

| **Methods** | **OSS** | **CC_OSS** | **CSS** | **Methods** | **OSS** | **CC_OSS** | **CSS** |
| --- | --- | --- | --- | --- | --- | --- | --- |
| HF/LanL2DZ | 4.66 | 9.31 | 89.75 | M06/ LanL2DZ | 1.49 | 2.98 | 20.99 |
| HF/DGDZVP | 25.39 | 50.79 | 56.02 | M06/ DGDZVP | 4.98 | 9.93 | 9.07 |
| HF/Def2TZVP | 23.77 | 46.52 | 51.15 | M06/ Def2TZVP | 4.31 | 8.62 | 58.23 |
| HF/6-31G* | 5.92 | 11.85 | 51.56 | M06/6-31G* | 2.62 | 5.25 | 51.51 |
| HF/6-311+G* | 5.81 | 11.62 | 51.57 | M06/6-311+G* | 3.66 | 7.32 | 58.38 |
| HF/6-311G** | 31.74 | 61.20 | 50.73 | M06/6-311G** | 2.31 | 4.62 | 48.25 |
| HF/cc-pVTZ | 5.83 | 11.67 | 50.82 | M06/cc-pVTZ | 4.27 | 8.54 | 58.20 |
| HF/aug-cc-pVTZ | 5.83 | 11.66 | 50.76 | M06/aug-cc-pVTZ | 4.44 | 8.88 | 59.38 |
| PBEPBE/LanL2DZ | 6.57 | 13.14 | 36.81 | MP2/ LanL2DZ | 4.29 | 8.59 | 81.51 |
| PBEPBE/DGDZVP | 5.44 | 10.88 | 78.62 | MP2/ DGDZVP | 6.91 | 13.82 | 45.94 |
| PBEPBE/Def2TZVP | 5.54 | 10.67 | 67.46 | MP2/ Def2TZVP | 19.23 | 37.64 | 38.57 |
| PBEPBE/6-31G* | 6.09 | 11.39 | 70.83 | MP2/6-31G* | 5.05 | 10.11 | 40.15 |
| PBEPBE/6-311+G* | 5.62 | 10.84 | 79.06 | MP2/6-311+G* | 4.73 | 9.48 | 39.24 |
| PBEPBE/6-311G** | 6.19 | 11.61 | 71.80 | MP2/6-311G** | 4.89 | 9.78 | 46.35 |
| PBEPBE/cc-pVTZ | 5.51 | 10.65 | 58.48 | MP2/cc-pVTZ | 4.40 | 8.80 | 37.45 |
| PBEPBE/aug-cc-pVTZ | 5.52 | 10.66 | 112.77 | MP2/aug-cc-pVTZ | 4.37 | 8.75 | 37.28 |
| B3LYP/LanL2DZ | 7.39 | 14.78 | 31.79 | CCSD(T)/LanL2DZ | 0.39 | 0.77 | 68.10 |
| B3LYP/DGDZVP | 3.90 | 7.80 | 36.37 | CCSD(T)/DGDZVP | 0.38 | 0.76 | 37.84 |
| B3LYP/Def2TZVP | 8.49 | 17.00 | 37.70 | CCSD(T)/Def2TZVP | 2.53 | 4.94 | 32.43 |
| B3LYP/6-31G* | 3.15 | 6.30 | 61.07 | CCSD(T)/6-31G* | 1.94 | 3.88 | 33.42 |
| B3LYP/6-311+G* | 10.07 | 19.72 | 37.82 | CCSD(T)/6-311+G* | 1.74 | 3.49 | 32.61 |
| B3LYP/6-311G** | 6.17 | 11.60 | 61.60 | CCSD(T)/6-311G** | 3.32 | 6.46 | 43.85 |
| B3LYP/cc-pVTZ | 8.06 | 16.14 | 37.57 | CCSD(T)/cc-pVTZ | 1.83 | 3.67 | 31.82 |
| B3LYP/aug-cc-pVTZ | 4.00 | 8.00 | 37.43 | CCSD(T)/aug-cc-pVTZ | 1.83 | 3.67 | 31.75 |
|  |  |  |  | **Exp** | - | **7.38** | - |

**Supplementary Table 1(B).** Relative energies (kcal/mol) calculated for singlet state (SS) of Sc^+^OH_2_ and HSc^+^OH complexes. Triplet state (TS) of Sc^+^OH_2_ and HSc^+^OH complexes is considered as a standard (energy zero) to calculate the relative energy.

| **Methods** | **Sc^+^OH_2_** | **HSc^+^OH** | **Methods** | **Sc^+^OH_2_** | **HSc^+^OH** |
| --- | --- | --- | --- | --- | --- |
| HF/LanL2DZ | 32.05 | -38.04 | M06/ LanL2DZ | -1.69 | -65.23 |
| HF/DGDZVP | 27.47 | -39.13 | M06/ DGDZVP | 0.05 | -65.32 |
| HF/Def2TZVP | 28.21 | -45.20 | M06/ Def2TZVP | -2.15 | -68.91 |
| HF/6-31G* | 28.11 | -43.47 | M06/6-31G* | -4.61 | -68.24 |
| HF/6-311+G* | 28.94 | -43.82 | M06/6-311+G* | -2.06 | -67.62 |
| HF/6-311G** | 27.10 | -44.49 | M06/6-311G** | -5.19 | -68.52 |
| HF/cc-pVTZ | 28.91 | -45.60 | M06/cc-pVTZ | -1.71 | -68.83 |
| HF/aug-cc-pVTZ | 28.84 | -45.52 | M06/aug-cc-pVTZ | -1.82 | -68.79 |
| PBEPBE/LanL2DZ | 17.45 | -57.15 | MP2/ LanL2DZ | 22.13 | -50.65 |
| PBEPBE/DGDZVP | 56.91 | -72.72 | MP2/ DGDZVP | 18.04 | -49.26 |
| PBEPBE/Def2TZVP | 81.20 | -70.74 | MP2/ Def2TZVP | 14.16 | -59.68 |
| PBEPBE/6-31G* | 94.41 | -61.75 | MP2/6-31G* | 14.94 | -52.30 |
| PBEPBE/6-311+G* | 78.48 | -59.55 | MP2/6-311+G* | 14.75 | -53.66 |
| PBEPBE/6-311G** | 20.63 | -61.97 | MP2/6-311G** | 18.52 | -59.00 |
| PBEPBE/cc-pVTZ | 22.08 | -73.75 | MP2/cc-pVTZ | 13.99 | -62.10 |
| PBEPBE/aug-cc-pVTZ | 26.98 | -60.27 | MP2/aug-cc-pVTZ | 13.92 | -62.29 |
| B3LYP/LanL2DZ | 13.66 | -63.33 | CCSD(T)/LanL2DZ | 7.92 | -53.17 |
| B3LYP/DGDZVP | 12.24 | -62.56 | CCSD(T)/DGDZVP | 7.44 | -52.49 |
| B3LYP/Def2TZVP | 11.31 | -65.24 | CCSD(T)/Def2TZVP | 5.78 | -63.51 |
| B3LYP/6-31G* | 9.13 | -64.70 | CCSD(T)/6-31G* | 6.04 | -55.27 |
| B3LYP/6-311+G* | 11.22 | -64.12 | CCSD(T)/6-311+G* | 5.99 | -56.88 |
| B3LYP/6-311G** | 9.00 | -64.93 | CCSD(T)/6-311G** | 10.35 | -62.62 |
| B3LYP/cc-pVTZ | 11.41 | -65.21 | CCSD(T)/cc-pVTZ | 6.19 | -65.90 |
| B3LYP/aug-cc-pVTZ | 11.40 | -65.16 | CCSD(T)/aug-cc-pVTZ | 6.31 | -65.97 |

**Supplementary Table 2.** List of the selected methods based on the calculated relative energies (kcal/mol) for open shell singlet (OSS), contamination corrected open shell singlet (CC_OSS), and close shell singlet (CSS) of bare Sc^+^ ion. Triplet state (TS) of bare Sc^+^ ion is considered as a standard (energy zero) to calculate the relative energy.

| **Methods** | **OSS** | **CC_OSS** | **CSS** |
| --- | --- | --- | --- |
| HF/LanL2DZ | 4.66 | 9.31 | 89.75 |
| B3LYP/DGDZVP | 3.90 | 7.80 | 36.37 |
| B3LYP/6-31G* | 3.15 | 6.30 | 61.07 |
| B3LYP/aug-cc-pVTZ | 4.00 | 8.00 | 37.43 |
| M06/ Def2TZVP | 4.31 | 8.62 | 58.23 |
| M06/6-31G* | 2.62 | 5.25 | 51.51 |
| M06/6-311+G* | 3.66 | 7.32 | 58.38 |
| M06/cc-pVTZ | 4.27 | 8.54 | 58.20 |
| M06/aug-cc-pVTZ | 4.44 | 8.88 | 59.38 |
| MP2/ LanL2DZ | 4.29 | 8.59 | 81.51 |
| MP2/6-311+G* | 4.73 | 9.48 | 39.24 |
| MP2/cc-pVTZ | 4.40 | 8.80 | 37.45 |
| MP2/aug-cc-pVTZ | 4.37 | 8.75 | 37.28 |
| CCSD(T)/6-311G** | 3.32 | 6.46 | 43.85 |
| **Exp** | - | **7.38** | - |

**Supplementary Table 3(A).** The variation of electron density ($\rho, in a.u.$), Laplacian of electron density ($\nabla^{2}\rho, in a.u.$), total energy density (H(**r**), $in a.u.$) and ration of potential and kinetic energy density ([-G(**r**)/V(**r**)]) as obtained at bond critical point of Sc-OH_2_, Sc-OH and Sc-H bonds of (ScOH_2_)_n_;n=1-2 ion- and their insertion complexes,obtained at MP2/6-31G* optimized geometry.

| **Sc^+^-OH_2_** | **Spin State** | **ρ** | **∇^2^ρ** | **H(r)** | **[-G(r)/V(r)]** |
| --- | --- | --- | --- | --- | --- |
| Sc^+^OH_2_ | TS | 0.0390 | 0.2559 | 0.0069 | 1.1385 |
| Sc^+^OH_2_ | SS | 0.0656 | 0.3404 | 0.0021 | 1.0254 |
| Sc^+^(OH_2_)_2_ | TS | 0.0385 | 0.2549 | 0.0069 | 1.1389 |
| Sc^+^(OH_2_)_2_ | SS | 0.0602 | 0.3201 | 0.0035 | 1.0482 |
| HSc^+^OH(OH_2_) | TS | 0.0439 | 0.2413 | 0.0055 | 1.1126 |
| HSc^+^OH(OH_2_) | SS | 0.0492 | 0.2793 | 0.0063 | 1.1095 |
| **Sc^+^-OH** |  |  |  |  |  |
| H-Sc^+^-OH | TS | 0.1620 | 0.8317 | -0.0645 | 0.8536 |
| H-Sc^+^-OH | SS | 0.1480 | 0.8097 | -0.0470 | 0.8229 |
| HSc^+^OH(OH_2_) | TS | 0.1231 | 0.7189 | -0.0258 | 0.8618 |
| HSc^+^OH(OH_2_) | SS | 0.1366 | 0.7779 | -0.0361 | 0.8408 |
| H_2_Sc^+^(OH)_2_ | TS | 0.1334 | 0.7665 | -0.0337 | 0.8489 |
| H_2_Sc^+^(OH)_2_ | SS | 0.1366 | 0.7779 | -0.0361 | 0.8504 |
| **Sc^+^-H** |  |  |  |  |  |
| H-Sc^+^-OH | TS | 0.0008 | 0.0006 | 0.0000 | 1.1850 |
| H-Sc^+^-OH | SS | 0.0865 | 0.0211 | -0.0301 | 0.5211 |
| HSc^+^OH(OH_2_) | TS | 0.0064 | 0.0196 | 0.0008 | 1.2660 |
| HSc^+^OH(OH_2_) | SS | 0.0806 | 0.0348 | -0.0271 | 0.5466 |
| H_2_Sc^+^(OH)_2_ | TS | 0.0062 | 0.0207 | 0.0010 | 1.2979 |
| H_2_Sc^+^(OH)_2_ | SS | 0.0140 | 0.0629 | 0.0038 | 1.4474 |
| **Sc^2+^-OH_2_** |  |  |  |  |  |
| Sc^2+^OH_2_ | DS | 0.0533 | 0.2625 | 0.0032 | 1.0531 |
| Sc^2+^(OH_2_)_2_ | DS | 0.0562 | 0.2799 | 0.0027 | 1.0416 |
| HSc^2+^OH(OH_2_) | DS | 0.0601 | 0.3283 | 0.0045 | 1.0614 |
| **Sc^2+^-OH** |  |  |  |  |  |
| H-Sc^2+^-OH | DS | 0.1745 | 0.9464 | -0.0732 | 0.8089 |
| HSc^2+^OH(OH_2_) | DS | 0.1648 | 0.9082 | -0.0627 | 0.8220 |
| H_2_Sc^2+^(OH)_2_ | DS | 0.1667 | 0.9152 | -0.0648 | 0.8192 |
| **Sc^2+^-H** |  |  |  |  |  |
| H-Sc^2+^-OH | DS | 0.0108 | 0.0251 | 0.0007 | 1.1425 |
| HSc^2+^OH(OH_2_) | DS | 0.0095 | 0.0237 | 0.0008 | 1.1907 |
| H_2_Sc^2+^(OH)_2_ | DS | 0.0096 | 0.0242 | 0.0008 | 1.1932 |
| **Sc^3+^-OH_2_** |  |  |  |  |  |
| Sc^3+^OH_2_ | SS | 0.1006 | 0.4518 | -0.0132 | 0.9051 |
| Sc^3+^(OH_2_)_2_ | SS | 0.0839 | 0.4059 | -0.0039 | 0.9647 |

**Supplementary Table 3(B).** The absolute value of kinetic energy density (G(**r**)$in a.u.$), potential energy density (V(**r**)$in a.u.$), and the percentage contribution of G(**r**) into total energy density (H(**r**)) as obtained at bond critical point of Sc-OH_2_, Sc-OH and Sc-H bonds of (ScOH_2_)_n_;n=1-2 ion- and their insertion complexes,obtainedat MP2/6-31G* level of theory.

| **Sc^+^-OH2** | **Spin State** | **G(r) in a.u** | **V(r) in a.u** | **G(r) in %** |
| --- | --- | --- | --- | --- |
| Sc^+^OH_2_ | TS | 0.0570 | 0.0501 | 53.24 |
| Sc^+^OH_2_ | OSS | 0.0570 | 0.0501 | 53.22 |
| Sc^+^OH_2_ | CSS | 0.0830 | 0.0809 | 50.63 |
| Sc^+^(OH_2_)_2_ | TS | 0.0568 | 0.0499 | 53.25 |
| Sc^+^(OH_2_)_2_ | OSS | 0.0567 | 0.0499 | 53.20 |
| Sc^+^(OH_2_)_2_ | CSS | 0.0764 | 0.0729 | 51.18 |
| HSc^+^OH(OH_2_) | TS | 0.0548 | 0.0492 | 52.66 |
| HSc^+^OH(OH_2_) | CSS | 0.0636 | 0.0573 | 52.60 |
| **Sc^+^-OH** |  |  |  |  |
| H-Sc^+^-OH | TS | 0.2724 | 0.3369 | 44.71 |
| H-Sc^+^-OH | CSS | 0.2494 | 0.2964 | 45.70 |
| HSc^+^OH(OH_2_) | TS | 0.2055 | 0.2314 | 47.04 |
| HSc^+^OH(OH_2_) | CSS | 0.2306 | 0.2667 | 46.37 |
| H_2_Sc^+^(OH)_2_ | TS | 0.2253 | 0.2590 | 46.52 |
| H_2_Sc^+^(OH)_2_ | CSS | 0.2306 | 0.2667 | 46.37 |
| **Sc^+^-H** |  |  |  |  |
| H-Sc^+^-OH | TS | 0.0002 | 0.0002 | 46.87 |
| H-Sc^+^-OH | CSS | 0.0354 | 0.0656 | 35.08 |
| HSc^+^OH(OH_2_) | TS | 0.0041 | 0.0032 | 55.81 |
| HSc^+^OH(OH_2_) | CSS | 0.0358 | 0.0629 | 36.27 |
| H_2_Sc^+^(OH)_2_ | TS | 0.0041 | 0.0031 | 57.13 |
| H_2_Sc^+^(OH)_2_ | CSS | 0.0120 | 0.0082 | 59.29 |
| **Sc^2+^-OH_2_** |  |  |  |  |
| Sc^2+^OH_2_ | DS | 0.0625 | 0.0593 | 51.29 |
| Sc^2+^(OH_2_)_2_ | DS | 0.0673 | 0.0646 | 51.02 |
| HSc^2+^OH(OH_2_) | DS | 0.0776 | 0.0731 | 51.49 |
| **Sc^2+^-OH** |  |  |  |  |
| H-Sc^2+^-OH | DS | 0.3098 | 0.3830 | 44.72 |
| HSc^2+^OH(OH_2_) | DS | 0.2898 | 0.3525 | 45.12 |
| H_2_Sc^2+^(OH)_2_ | DS | 0.2936 | 0.3584 | 45.03 |
| **Sc^2+^-H** |  |  |  |  |
| H-Sc^2+^-OH | DS | 0.0056 | 0.0049 | 53.33 |
| HSc^2+^OH(OH_2_) | DS | 0.0051 | 0.0043 | 54.35 |
| H_2_Sc^2+^(OH)_2_ | DS | 0.0052 | 0.0044 | 54.40 |
| **Sc^3+^-OH_2_** |  |  |  |  |
| Sc^3+^OH_2_ | SS | 0.1262 | 0.1394 | 47.51 |
| Sc^3+^(OH_2_)_2_ | SS | 0.1053 | 0.1092 | 49.10 |

**Supplementary Table 4.**Sequential binding energy (${\Delta E}_{\mathrm{seq}}$; kcal/mol) of mono-cationic singlet state (MSS), and triplet state (MTS), di-cationic doublet state (DDS), and tri-cationic singlet state (TSS) of Sc ion in Sc^z+^(OH_2_)_n_; z = 1-3 and n = 1-2 complexes using MP2/6-31G* optimized geometry.

| **Methods** | **MSS** | | **MTS** | | **DDS** | | **TSS** | |
| --- | --- | --- | --- | --- | --- | --- | --- | --- |
|  | **n = 1** | **n = 2** | **n = 1** | **n = 2** | **n = 1** | **n = 2** | **n = 1** | **n = 2** |
| HF/LanL2DZ | -98.68 | -50.24 | -41.08 | -35.93 | -179.81 | -60.07 | -153.18 | -124.68 |
| B3LYP/DGDZVP | -61.45 | -43.12 | -37.19 | -33.18 | -75.93 | -53.01 | -165.74 | -123.34 |
| B3LYP/6-31G* | -93.66 | -47.29 | -41.49 | -35.98 | -66.04 | -65.26 | -176.28 | -128.35 |
| B3LYP/aug-cc-pVTZ | -59.95 | -39.47 | -33.73 | -29.47 | -58.00 | -57.32 | -165.00 | -119.15 |
| M06/ Def2TZVP | -97.21 | -42.19 | -36.61 | -31.29 | -56.29 | -42.31 | -161.51 | -118.17 |
| M06/6-31G* | -98.56 | -47.61 | -42.21 | -35.72 | -74.82 | -49.81 | -170.70 | -125.18 |
| M06/6-311+G* | -100.61 | -45.33 | -40.08 | -34.54 | -73.06 | -46.82 | -163.67 | -121.45 |
| M06/cc-pVTZ | -98.02 | -43.45 | -37.86 | -32.54 | -57.82 | -44.20 | -162.78 | -119.33 |
| M06/aug-cc-pVTZ | -96.73 | -41.02 | -35.30 | -30.10 | -68.12 | -41.87 | -161.03 | -117.10 |
| MP2/ LanL2DZ | -101.54 | -52.20 | -42.37 | -37.78 | -180.35 | -60.88 | -158.36 | -124.20 |
| MP2/6-311+G* | -59.56 | -45.61 | -34.98 | -30.95 | -132.55 | -58.39 | -145.82 | -116.72 |
| MP2/cc-pVTZ | -55.69 | -42.38 | -31.94 | -28.12 | -126.77 | -54.22 | -141.14 | -112.08 |
| MP2/aug-cc-pVTZ | -59.26 | -49.66 | -28.76 | -25.23 | -123.36 | -50.97 | -137.67 | -108.51 |
| CCSD(T)/6-311G** | -64.97 | -38.89 | -31.12 | -29.19 | -128.86 | -56.20 | -141.43 | -113.13 |

**Supplementary Table 5(A).**Interaction energy (IE; kcal/mol) and nuclear repulsion energy (Hartree) of singlet Sc^+^(OH_2_)_n_; n=1-6 complexes obtained at CCSD(T)/6-311G**//MP2/6-31G* level of theory.

| **No. of Water** | **Structures (X+Y)** | **Nuclear Repulsion Energy** | **Interaction Energy** | | | | | |
| --- | --- | --- | --- | --- | --- | --- | --- | --- |
|  |  |  | **HF** | **MP2** | **MP3** | **MP4D** | **CCSD** | **CCSD(T)** |
| n = 1 | 1+0 | 58.86434012 | -53.66 | -59.37 | -60.19 | -61.07 | -63.99 | **-64.96** |
| n = 2 | 2+0 | 128.04481871 | -95.28 | -103.28 | -103.23 | -103.69 | -104.23 | -105.41 |
|  | 1+1 | 116.28363845 | -75.58 | -81.73 | -81.87 | -82.54 | -84.44 | **-85.52** |
| n = 3 | 3+0 | 213.54402482 | -117.15 | -131.24 | -130.89 | -131.67 | -131.97 | -134.50 |
|  | 2+1 | 192.31076561 | -113.29 | -121.75 | -121.34 | -121.71 | -122.00 | -123.29 |
|  | 1+2 | 184.24131194 | -94.14 | -100.61 | -100.21 | -100.73 | -102.01 | **-103.20** |
| n = 4 | 4+0 | 316.44033816 | -145.08 | -161.69 | -160.68 | -161.17 | -160.90 | -163.89 |
|  | 3+1 | 286.98806881 | -133.71 | -146.59 | -145.74 | -146.15 | -146.11 | -148.23 |
|  | 2+2 | 261.75514557 | -129.94 | -138.72 | -138.02 | -138.31 | -138.42 | -139.77 |
| n = 5 | 5+0 | 433.38960279 | -148.81 | -171.45 | -169.78 | -170.62 | -170.22 | -174.51 |
|  | 4+1 | 411.09934080 | -158.61 | -175.46 | -174.02 | -174.48 | -174.46 | -177.62 |
|  | 3+2 | 368.27509716 | -147.90 | -161.42 | -160.27 | -160.64 | -160.51 | -162.77 |
|  | 2+3 | 342.77539090 | -143.02 | -152.30 | -151.25 | -151.47 | -151.37 | -152.86 |
| n = 6 | 6+0 | 565.14462918 | -170.42 | -198.63 | -195.85 | -196.80 | -195.52 | -201.11 |
|  | 5+1 | 548.88186194 | -158.17 | -181.64 | -179.63 | -180.46 | -180.04 | -184.60 |
|  | 4+2 | 500.52021388 | -170.49 | -187.97 | -186.29 | -186.72 | -186.58 | -189.81 |
|  | 3+3 | 536.47837222 | -172.65 | -192.90 | -191.11 | -191.52 | -191.00 | -194.76 |
|  | 2+4 | 430.92784008 | -156.15 | -165.79 | -164.49 | -164.66 | -164.44 | -166.01 |

**Supplementary Table 5(B).**Interaction energy (IE; kcal/mol) and nuclear repulsion energy (Hartree) of triplet Sc^+^(OH_2_)_n_; n=1-6 complexes obtained at CCSD(T)/6-311G**// MP2/6-31G* level of theory.

| **No. of Water** | **Structures (X+Y)** | **Nuclear Repulsion Energy** | **Interaction Energy** | | | | | |
| --- | --- | --- | --- | --- | --- | --- | --- | --- |
|  |  |  | **HF** | **MP2** | **MP3** | **MP4D** | **CCSD** | **CCSD(T)** |
| n = 1 | 1+0 | 55.85907248 | -29.69 | -31.20 | -30.77 | -30.75 | -30.99 | -31.13 |
| n = 2 | 2+0 | 122.56249203 | -58.02 | -61.40 | -60.67 | -60.63 | -61.06 | -61.43 |
|  | 1+1 | 111.92628743 | -45.65 | -47.55 | -46.94 | -46.93 | -47.23 | -47.43 |
| n = 3 | 3+0 | 210.18679195 | -77.94 | -82.28 | -81.04 | -80.83 | -81.32 | -81.89 |
|  | 2+1 | 187.82654574 | -71.70 | -75.46 | -74.55 | -74.48 | -74.93 | -75.40 |
|  | 1+2 | 177.67593114 | -59.95 | -62.12 | -61.35 | -61.33 | -61.68 | -61.93 |
| n = 4 | 4+0 | 304.50967492 | -103.06 | -109.00 | -107.15 | -106.92 | -107.45 | -108.19 |
|  | 3+1 | 283.44112651 | -94.55 | -98.43 | -97.03 | -96.75 | -97.12 | -97.67 |
|  | 2+2 | 256.90915011 | -84.72 | -88.86 | -87.83 | -87.77 | -88.25 | -88.76 |
| n = 5 | 5+0 | 416.58317487 | -120.30 | -127.65 | -125.31 | -125.00 | -125.68 | -126.72 |
|  | 4+1 | 401.10944208 | -116.97 | -122.58 | -120.56 | -120.30 | -120.90 | -121.65 |
|  | 3+2 | 374.85758679 | -107.38 | -111.57 | -110.07 | -109.88 | -110.50 | -111.05 |
|  | 2+3 | 337.29269448 | -94.18 | -98.08 | -96.98 | -96.85 | -97.27 | -97.77 |
| n = 6 | 6+0 | 545.71476808 | -139.63 | -150.91 | -147.48 | -147.10 | -147.66 | -149.26 |
|  | 5+1 | 535.20026663 | -120.78 | -127.67 | -125.42 | -125.10 | -125.89 | -126.99 |
|  | 4+2 | 502.46562878 | -129.72 | -135.59 | -133.46 | -133.09 | -133.68 | -134.60 |
|  | 3+3 | 475.58095999 | -120.12 | -123.58 | -121.91 | -121.61 | -122.31 | -122.87 |
|  | 2+4 | 433.47069207 | -111.05 | -114.62 | -113.14 | -113.01 | -113.54 | -113.96 |

**Supplementary Table 5(C).**Interaction energy (IE; kcal/mol) and nuclear repulsion energy (Hartree) of doublet Sc^2+^(OH_2_)_n_; n=1-6 complexes obtained at CCSD(T)/6-311G**//MP2/6-31G* level of theory.

| **No. of Water** | **Structures (X+Y)** | **Nuclear Repulsion Energy** | **Interaction Energy** | | | | | |
| --- | --- | --- | --- | --- | --- | --- | --- | --- |
|  |  |  | **HF** | **MP2** | **MP3** | **MP4D** | **CCSD** | **CCSD(T)** |
| n = 1 | 1+0 | 55.94995685 | -128.81 | -129.49 | -128.71 | -128.64 | -128.82 | -128.85 |
| n = 2 | 2+0 | 125.38596309 | -185.01 | -186.89 | -185.51 | -185.37 | -185.70 | -185.92 |
|  | 1+1 | 115.58945923 | -166.28 | -167.85 | -166.65 | -166.62 | -166.96 | -167.20 |
| n = 3 | 3+0 | 213.18641877 | -257.74 | -258.84 | -256.93 | -256.53 | -256.94 | -257.15 |
|  | 2+1 | 193.80998239 | -220.42 | -223.20 | -221.49 | -221.37 | -221.81 | -222.25 |
|  | 1+2 | 185.06671782 | -194.73 | -197.15 | -195.48 | -195.49 | -196.04 | -196.51 |
| n = 4 | 4+0 | 306.40635968 | -306.04 | -306.68 | -304.57 | -304.05 | -304.56 | -304.64 |
|  | 3+1 | 290.55924459 | -289.60 | -291.12 | -288.97 | -288.55 | -289.04 | -289.37 |
|  | 2+2 | 268.80323563 | -268.42 | -269.19 | -267.34 | -267.02 | -267.35 | -267.53 |
| n = 5 | 5+0 | 426.48968737 | -347.55 | -346.79 | -344.72 | -343.89 | -344.38 | -344.37 |
|  | 4+1 | 409.91361953 | -333.19 | -333.79 | -331.68 | -331.03 | -331.57 | -331.79 |
|  | 3+2 | 369.65516524 | -317.20 | -319.00 | -316.61 | -316.22 | -316.78 | -317.13 |
|  | *2+3 | 370.13259959 | -299.53 | -300.88 | -298.74 | -298.40 | -298.89 | -299.28 |
| n = 6 | 6+0 | 543.44456533 | -384.64 | -383.14 | -381.02 | -380.07 | -380.59 | -380.44 |
|  | 5+1 | 528.70833114 | -371.83 | -371.08 | -368.68 | -367.83 | -368.40 | -368.45 |
|  | 4+2 | 514.52264137 | -359.21 | -359.91 | -357.62 | -356.93 | -357.55 | -357.82 |
|  | 3+3 | 461.88333733 | -343.65 | -345.64 | -343.05 | -342.63 | -343.26 | -343.66 |
|  | 2+4 | 437.27975537 | -300.24 | -304.07 | -301.51 | -301.36 | -302.03 | -302.72 |

**Supplementary Table 5(D).**Interaction energy (IE; kcal/mol) and nuclear repulsion energy (Hartree) of singlet Sc^3+^(OH_2_)_n_; n=1-6 complexes obtained at CCSD(T)/6-311G**// MP2/6-31G* level of theory.

| **No. of Water** | **Structures (X+Y)** | **Nuclear Repulsion Energy** | **Interaction Energy** | | | | | |
| --- | --- | --- | --- | --- | --- | --- | --- | --- |
|  |  |  | **HF** | **MP2** | **MP3** | **MP4D** | **CCSD** | **CCSD(T)** |
| n = 1 | 1+0 | 62.10148125 | -139.58 | -142.97 | -139.41 | -139.70 | -140.31 | -141.40 |
| n = 2 | 2+0 | 132.52537759 | -248.61 | -251.62 | -247.48 | -247.42 | -248.06 | -248.98 |
|  | 1+1 | 121.68369514 | -196.09 | -203.17 | -198.09 | -198.81 | -199.92 | -202.09 |
| n = 3 | 3+0 | 221.94609312 | -352.60 | -355.59 | -350.61 | -350.28 | -351.05 | -352.03 |
|  | 2+1 | 203.09293935 | -300.99 | -306.59 | -301.17 | -301.37 | -302.37 | -304.00 |
|  | 1+2 | 189.91580592 | -246.86 | -256.48 | -249.77 | -250.79 | -252.24 | -242.31 |
| n = 4 | 4+0 | 324.55509650 | -441.74 | -443.97 | -438.65 | -438.01 | -438.86 | -439.73 |
|  | 3+1 | 297.23890679 | -392.40 | -397.11 | -391.47 | -391.28 | -392.22 | -393.57 |
|  | 2+2 | 261.35932414 | -330.48 | -337.44 | -331.79 | -332.19 | -333.28 | -335.01 |
| n = 5 | 5+0 | 443.17212183 | -513.35 | -513.87 | -508.86 | -507.87 | -508.69 | -509.26 |
|  | 4+1 | 424.86491032 | -492.67 | -495.55 | -489.81 | -489.16 | -490.13 | -491.22 |
|  | 3+2 | 377.21637857 | -425.04 | -431.02 | -424.97 | -424.90 | -425.94 | -427.47 |
|  | 2+3 | 343.10873781 | -366.14 | -374.75 | -368.17 | -368.71 | -370.05 | -372.26 |
| n = 6 | 6+0 | 576.08204635 | -580.29 | -579.04 | -574.33 | -572.99 | -573.76 | -574.03 |
|  | 5+1 | 550.97602263 | -561.73 | -562.78 | -557.38 | -556.37 | -557.31 | -558.04 |
|  | 4+2 | 512.98012840 | -498.17 | -503.02 | -496.99 | -496.60 | -497.63 | -498.96 |
|  | 3+3 | 467.60467776 | -454.23 | -461.99 | -455.27 | -455.45 | -456.73 | -458.62 |
|  | 2+4 | 430.92784008 | -399.44 | -408.96 | -401.79 | -402.39 | -403.84 | -406.28 |

**Supplementary Table 6.**Variation in average M-O distance (Å) of mono-cationic singlet state (MSS), and triplet state (MTS), di-cationic doublet state (DDS), and tri-cationic singlet state (TSS) of Sc(OH_2_)_n_; n=1-6 complexes, obtained at MP2/6-31G* level of theory.

| **No. of Water** | **Structures (X+Y)** | **MSS** | **MTS** | **DDS** | **TSS** |
| --- | --- | --- | --- | --- | --- |
| n=1 | 1+0 | 2.118 | 2.260 | 2.250 | 1.960 |
| n = 2 | 2+0 | 2.149 | 2.267 | 2.100 | 2.045 |
|  | 1+1 | 2.053 | 2.201 | 2.127 | 1.903 |
| n = 3 | 3+0 | 2.166 | 2.262 | 2.167 | 2.062 |
|  | 2+1 | 2.127 | 2.202 | 2.145 | 2.000 |
|  | 1+2 | 2.205 | 2.350 | 2.254 | 1.860 |
| n = 4 | 4+0 | 2.187 | 2.219 | 2.146 | 2.094 |
|  | 3+1 | 2.107 | 2.186 | 2.117 | 2.049 |
|  | 2+2 | 2.011 | 2.160 | 2.058 | 2.105 |
| n = 5 | 5+0 | 2.275 | 2.376 | 2.239 | 2.139 |
|  | 4+1 | 2.205 | 2.334 | 2.189 | 2.082 |
|  | 3+2 | 2.171 | 2.301 | 2.172 | 2.073 |
|  | 2+3 | 2.089 | 2.162 | 2.122 | 2.090 |
| n = 6 | 6+0 | 2.298 | 2.362 | 2.332 | 2.172 |
|  | 5+1 | 2.273 | 2.376 | 2.244 | 2.128 |
|  | 4+2 | 2.200 | 2.279 | 2.175 | 2.118 |
|  | 3+3 | 2.118 | 2.236 | 2.156 | 2.130 |
|  | 2+4 | 2.075 | 2.213 | 2.120 | 2.074 |

**Supplementary Table 7(A).** The variation of electron density (ρ in a.u.), Laplacian of electron density (∇^2^ρ in a.u.), total energy density (H(**r**) in a.u.) and ration of kinetic and potential energy density ([-G(r)/V(r)]), obtained at bond critical point of Sc^+^-OH_2_ bonds of singlet Sc^+^(OH_2_)_n_; n=1-6 complexes, calculated at MP2/6-31G* level of theory.

| **No. of Water** | **Structures (X+Y)** | **ρ** | **∇^2^ρ** | **H(r)** | **[-G(r)/[V(r)]** |
| --- | --- | --- | --- | --- | --- |
| n = 1 | 1+0 | 0.0656 | 0.3404 | 0.0021 | 1.0254 |
| n = 2 | 2+0 | 0.0602 | 0.3201 | 0.0035 | 1.0482 |
|  | 1+1 | 0.0792 | 0.3906 | -0.0033 | 0.9687 |
| n = 3 | 3+0 | 0.0526 | 0.2986 | 0.0063 | 1.1010 |
|  | 2+1 | 0.0646 | 0.3386 | 0.0021 | 1.0299 |
|  | 1+2 | 0.0895 | 0.4242 | -0.0086 | 0.9303 |
| n = 4 | 4+0 | 0.0488 | 0.2680 | 0.0059 | 1.1068 |
|  | 3+1 | 0.0541 | 0.2902 | 0.0038 | 1.0707 |
|  | 2+2 | 0.0683 | 0.3551 | 0.0012 | 1.0134 |
| n = 5 | 5+0 | 0.0424 | 0.2273 | 0.0049 | 1.1064 |
|  | 4+1 | 0.0499 | 0.2679 | 0.0053 | 1.0938 |
|  | 3+2 | 0.0564 | 0.3010 | 0.0037 | 1.0615 |
|  | 2+3 | 0.0722 | 0.3724 | -0.0003 | 0.9985 |
| n = 6 | 6+0 | 0.0405 | 0.2088 | 0.0046 | 1.1081 |
|  | 5+1 | 0.0440 | 0.2283 | 0.0044 | 1.0939 |
|  | 4+2 | 0.0510 | 0.2711 | 0.0049 | 1.0863 |
|  | 3+3 | 0.0438 | 0.2228 | 0.0046 | 1.0993 |
|  | 2+4 | 0.0751 | 0.3829 | -0.0013 | 0.9868 |

**Supplementary Table 7(B).**The variation of electron density (ρ in a.u.), Laplacian of electron density (∇^2^ρ in a.u.), total energy density (H(**r**) in a.u.) and ration of kinetic and potential energy density ([-G(r)/V(r)]), obtained at bond critical point of Sc^+^-OH_2_ bonds of triplet Sc^+^(OH_2_)_n_; n=1-6 complexes, calculated at MP2/6-31G* level of theory.

| **No. of Water** | **Structures (X+Y)** | **ρ** | **∇^2^ρ** | **H(r)** | **[-G(r)/[V(r)]** |
| --- | --- | --- | --- | --- | --- |
| n = 1 | 1+0 | 0.0390 | 0.2559 | 0.0069 | 1.1385 |
| n = 2 | 2+0 | 0.0385 | 0.2549 | 0.0069 | 1.1389 |
|  | 1+1 | 0.0464 | 0.3019 | 0.0069 | 1.1115 |
| n = 3 | 3+0 | 0.0396 | 0.2532 | 0.0069 | 1.1430 |
|  | 2+1 | 0.0460 | 0.3093 | 0.0074 | 1.1194 |
|  | 1+2 | 0.0525 | 0.3375 | 0.0063 | 1.0879 |
| n = 4 | 4+0 | 0.0328 | 0.1889 | 0.0053 | 1.1462 |
|  | 3+1 | 0.0418 | 0.2492 | 0.0064 | 1.1323 |
|  | 2+2 | 0.0483 | 0.3225 | 0.0072 | 1.1092 |
| n = 5 | 5+0 | 0.0312 | 0.1778 | 0.0048 | 1.1389 |
|  | 4+1 | 0.0346 | 0.1943 | 0.0051 | 1.1334 |
|  | 3+2 | 0.0374 | 0.2110 | 0.0056 | 1.1368 |
|  | 2+3 | 0.0520 | 0.3468 | 0.0069 | 1.0962 |
| n = 6 | 6+0 | 0.0327 | 0.1824 | 0.0048 | 1.1321 |
|  | 5+1 | 0.0321 | 0.1771 | 0.0043 | 1.1208 |
|  | 4+2 | 0.0404 | 0.2297 | 0.0053 | 1.1129 |
|  | 3+3 | 0.0442 | 0.2667 | 0.0054 | 1.0996 |
|  | 2+4 | 0.0460 | 0.2837 | 0.0063 | 1.1089 |

**Supplementary Table 7(C).**The variation of electron density (ρ in a.u.), Laplacian of electron density (∇^2^ρ in a.u.), total energy density (H(**r**) in a.u.) and ration of kinetic and potential energy density ([-G(r)/V(r)]), obtained at bond critical point of Sc^2+^-OH_2_ bonds of doublet Sc^2+^(OH_2_)_n_; n=1-6 complexes, calculated at MP2/6-31G* level of theory.

| **No. of Water** | **Structures (X+Y)** | **ρ** | **∇^2^ρ** | **H(r)** | **[-G(r)/[V(r)]** |
| --- | --- | --- | --- | --- | --- |
| n = 1 | 1+0 | 0.0531 | 0.2615 | 0.0032 | 1.0534 |
| n = 2 | 2+0 | 0.0573 | 0.2870 | 0.0026 | 1.0385 |
|  | 1+1 | 0.0719 | 0.3581 | -0.0016 | 0.9827 |
| n = 3 | 3+0 | 0.0544 | 0.2995 | 0.0050 | 1.0772 |
|  | 2+1 | 0.0658 | 0.3298 | 0.0000 | 1.0078 |
|  | 1+2 | 0.0858 | 0.4149 | -0.0081 | 0.9326 |
| n = 4 | 4+0 | 0.0413 | 0.2405 | 0.0062 | 1.1288 |
|  | 3+1 | 0.0580 | 0.3192 | 0.0041 | 1.0626 |
|  | 2+2 | 0.0607 | 0.3477 | 0.0048 | 1.0620 |
| n = 5 | 5+0 | 0.0412 | 0.2482 | 0.0066 | 1.1360 |
|  | 4+1 | 0.0514 | 0.2828 | 0.0053 | 1.0890 |
|  | 3+2 | 0.0547 | 0.2946 | 0.0043 | 1.0686 |
|  | 2+3 | 0.0784 | 0.3984 | 0.1995 | 0.9990 |
| n = 6 | 6+0 | 0.0329 | 0.1989 | 0.0060 | 1.1605 |
|  | 5+1 | 0.0410 | 0.2618 | 0.0071 | 1.1391 |
|  | 4+2 | 0.0537 | 0.2940 | 0.0049 | 1.0795 |
|  | 3+3 | 0.0569 | 0.3065 | 0.0041 | 1.0596 |
|  | 2+4 | 0.0705 | 0.3420 | -0.0015 | 0.9826 |

**Supplementary Table 7(D).**The variation of electron density (ρ in a.u.), Laplacian of electron density (∇^2^ρ in a.u.), total energy density (H(**r**) in a.u.) and ration of kinetic and potential energy density ([-G(r)/V(r)]), obtained at bond critical point of Sc^3+^-OH_2_ bonds of singlet Sc^3+^(OH_2_)_n_; n=1-6 complexes, calculated at MP2/6-31G* level of theory.

| **No. of Water** | **Structures (X+Y)** | **ρ** | **∇^2^ρ** | **H(r)** | **[-G(r)/[V(r)]** |
| --- | --- | --- | --- | --- | --- |
| n = 1 | 1+0 | 0.1007 | 0.4522 | -0.0133 | 0.9048 |
| n = 2 | 2+0 | 0.0790 | 0.3947 | -0.0020 | 0.9809 |
|  | 1+1 | 0.1197 | 0.5080 | -0.0273 | 0.8497 |
| n = 3 | 3+0 | 0.0734 | 0.3742 | 0.0006 | 1.0063 |
|  | 2+1 | 0.0904 | 0.4304 | -0.0077 | 0.9373 |
|  | 1+2 | 0.1360 | 0.5510 | -0.0416 | 0.8118 |
| n = 4 | 4+0 | 0.0664 | 0.3460 | 0.0029 | 1.0363 |
|  | 3+1 | 0.0768 | 0.3899 | -0.0011 | 0.9885 |
|  | 2+2 | 0.0707 | 0.3254 | -0.0010 | 0.9883 |
| n = 5 | 5+0 | 0.0592 | 0.3092 | 0.0043 | 1.0624 |
|  | 4+1 | 0.0692 | 0.3591 | 0.0018 | 1.0238 |
|  | 3+2 | 0.0732 | 0.3624 | -0.0003 | 0.9965 |
|  | 2+3 | 0.0747 | 0.4542 | 0.0331 | 1.5724 |
| n = 6 | 6+0 | 0.0542 | 0.2837 | 0.0050 | 1.0813 |
|  | 5+1 | 0.0610 | 0.3181 | 0.0038 | 1.0548 |
|  | 4+2 | 0.0663 | 0.3232 | 0.0008 | 1.0103 |
|  | 3+3 | 0.0661 | 0.3090 | -0.0001 | 0.9989 |
|  | 2+4 | 0.0765 | 0.3582 | -0.0029 | 0.9692 |

**Supplementary Table 8(A).**NPA charge on Sc ion and interaction energy (IE in kcal/mol) of mono-cationic singlet state (MSS), and triplet state (MTS), di-cationic doublet state (DDS), and tri-cationic singlet state (TSS) of Sc^z+^(OH_2_)_n_; z = 1-3 and n = 1-6 complexes, obtained at CCSD(T)/6-311G**//MP2/6-31G* level of theory.

| **No. of Water** | **Structures (X+Y)** | **MSS** | | **MTS** | | **DDS** | | **TSS** | |
| --- | --- | --- | --- | --- | --- | --- | --- | --- | --- |
|  |  | **Charge** | **IE** | **Charge** | **IE** | **Charge** | **IE** | **Charge** | **IE** |
| n=1 | 1+0 | 0.870 | -64.96 | 0.894 | -31.13 | 1.859 | -128.85 | 2.864 | -141.40 |
| n = 2 | 2+0 | 0.754 | -105.41 | 0.792 | -61.43 | 1.719 | -185.92 | 2.810 | -248.98 |
|  | 1+1 | 0.825 | -85.52 | 0.86 | -47.43 | 1.813 | -167.20 | 2.811 | -202.09 |
| n = 3 | 3+0 | 0.653 | -134.50 | 0.724 | -81.89 | 1.643 | -257.15 | 2.673 | -352.03 |
|  | 2+1 | 0.722 | -123.29 | 0.764 | -75.40 | 1.702 | -222.25 | 2.733 | -304.00 |
|  | 1+2 | 0.788 | -103.20 | 0.830 | -61.93 | 1.772 | -196.51 | 2.765 | -242.31 |
| n = 4 | 4+0 | 0.563 | -163.89 | 0.640 | -108.19 | 1.561 | -304.64 | 2.551 | -439.73 |
|  | 3+1 | 0.632 | -148.23 | 0.687 | -97.67 | 1.618 | -289.37 | 2.643 | -393.57 |
|  | 2+2 | 0.688 | -139.77 | 0.735 | -88.76 | 1.666 | -267.53 | 2.789 | -335.01 |
| n = 5 | 5+0 | 0.509 | -174.51 | 0.566 | -126.72 | 1.500 | -344.37 | 2.479 | -509.26 |
|  | 4+1 | 0.556 | -177.62 | 0.626 | -121.65 | 1.546 | -331.79 | 2.530 | -491.22 |
|  | 3+2 | 0.539 | -162.77 | 0.704 | -111.05 | 1.531 | -317.13 | 2.656 | -427.47 |
|  | 2+3 | 0.098 | -152.86 | 0.074 | -97.77 | 1.430 | -299.28 | 2.765 | -372.26 |
| n = 6 | 6+0 | 0.431 | -201.11 | 0.531 | -149.26 | 1.450 | -380.44 | 2.399 | -574.03 |
|  | 5+1 | 0.425 | -184.60 | 0.544 | -126.99 | 1.472 | -368.45 | 2.457 | -558.04 |
|  | 4+2 | 0.480 | -189.81 | 0.608 | -134.60 | 1.470 | -357.82 | 2.558 | -498.96 |
|  | 3+3 | 0.614 | -194.76 | 0.683 | -122.87 | 1.552 | -343.66 | 2.671 | -458.62 |
|  | 2+4 | 0.613 | -166.01 | 0.705 | -113.96 | 1.625 | -302.72 | 2.751 | -406.28 |

**Supplementary Table 8(B).**Spin density on Sc ion of mono-cationic triplet state (MTS), and di-cationic doublet state (DDS) of Sc^z+^(OH_2_)_n_; z = 1-3 and n = 1-6 complexes, obtained at CCSD(T)/6-311G**//MP2/6-31G* level of theory.

| **Complex** | **MTS** | **MDS** |
| --- | --- | --- |
| 1+0 | 2.020 | 0.996 |
| 2+0 | 2.030 | 1.010 |
| 1+1 | 2.029 | 0.994 |
| 3+0 | 2.065 | 1.012 |
| 2+1 | 2.039 | 1.015 |
| 1+2 | 2.035 | 0.995 |
| 4+0 | 2.111 | 1.005 |
| 3+1 | 2.079 | 1.015 |
| 2+2 | 2.047 | 1.007 |
| 5+0 | 2.157 | 1.004 |
| 4+1 | 2.123 | 1.022 |
| 3+2 | 2.083 | 1.015 |
| 2+3 | 2.054 | 1.015 |
| 6+0 | 1.891 | 1.002 |
| 5+1 | 2.175 | 1.000 |
| 4+2 | 2.076 | 1.026 |
| 3+3 | 2.100 | 1.017 |
| 2+4 | 2.089 | 1.025 |

**Supplementary Table 9(A).** Contribution of energy components (kcal/mol) such as electrostatic (ELC), exchange (EX), repulsion (REP), polarization (POL), dispersion (DISP), and into the interaction energy of ground state Sc^+^(OH_2_)_n_; n=1-6 complexes obtained using LMO-EDA scheme at B3LYP/6-311G**//MP2/6-31G* for Hydrogen (H) and Oxygen (O) and B3LYP/cc-pVTZ//MP2/6-31G* for metal ion.

| **Energy components** | **1+0** | **2+0** | **3+0** | **4+0** | **5+0** | **6+0** |
| --- | --- | --- | --- | --- | --- | --- |
| ELC | -80.29 | -147.44 | -159.38 | -200.43 | -207.36 | -220.76 |
| EX | -44.14 | -81.83 | -64.34 | -90.22 | -95.70 | -97.57 |
| REP | 149.05 | 278.88 | 230.60 | 317.58 | 329.64 | 336.51 |
| POL | -65.28 | -126.93 | -111.96 | -155.17 | -159.73 | -172.76 |
| DISP | -4.63 | -9.84 | -15.26 | -18.37 | -20.78 | -23.24 |
|  | **1+1** | **1+2** | **2+1** | **2+2** | **2+3** | **2+4** |
| ELC | -110.10 | -105.57 | -150.42 | -130.18 | -164.67 | -174.27 |
| EX | -54.74 | -31.82 | -70.89 | -26.64 | -56.00 | -53.46 |
| REP | 186.48 | 116.68 | 242.36 | 108.64 | 200.27 | 194.03 |
| POL | -83.82 | -59.67 | -116.26 | -63.43 | -103.96 | -103.69 |
| DISP | -4.94 | -5.13 | -10.18 | -10.46 | -10.61 | -10.80 |
|  | **3+1** | **3+2** | **3+3** | **4+1** | **4+2** | **5+1** |
| ELC | -172.75 | -171.23 | -214.62 | -206.81 | -214.80 | -206.38 |
| EX | -73.26 | -57.75 | -92.09 | -86.98 | -85.64 | -85.81 |
| REP | 256.09 | 206.61 | 318.75 | 306.18 | 300.29 | 298.65 |
| POL | -125.39 | -107.55 | -167.50 | -153.85 | -153.11 | -149.42 |
| DISP | -14.47 | -14.75 | -20.06 | -18.02 | -18.10 | -20.84 |

**Supplementary Table 9(B).**Contribution of energy components (kcal/mol) such as electrostatic (ELC), exchange (EX), repulsion (REP), polarization (POL), dispersion (DISP), and into the interaction energy of tripletSc^+^(OH_2_)_n_; n=1-6 complexes obtained using LMO-EDA scheme at B3LYP/6-311G**//MP2/6-31G* for Hydrogen (H) and Oxygen (O) and B3LYP/cc-pVTZ//MP2/6-31G* for metal ion.

| **Energy components** | **1+0** | **2+0** | **3+0** | **4+0** | **5+0** | **6+0** |
| --- | --- | --- | --- | --- | --- | --- |
| ELC | -42.04 | -80.34 | -152.39 | -158.20 | -171.93 | -212.17 |
| EX | -8.56 | -16.25 | -77.96 | -65.06 | -75.45 | -88.44 |
| REP | 35.07 | 66.84 | 273.18 | 220.85 | 261.89 | 302.77 |
| POL | -20.79 | -37.40 | -128.32 | -115.71 | -129.71 | -161.44 |
| DISP | -3.68 | -8.44 | -13.37 | -13.78 | -18.43 | -22.51 |
|  | **1+1** | **1+2** | **2+1** | **2+2** | **2+3** | **2+4** |
| ELC | -71.57 | -98.65 | -122.84 | -154.72 | -170.94 | -144.50 |
| EX | -27.12 | -39.36 | -55.32 | -67.91 | -71.95 | -41.20 |
| REP | 91.84 | 135.56 | 193.13 | 244.96 | 261.04 | 146.56 |
| POL | -47.04 | -67.06 | -97.10 | -118.25 | -127.28 | -78.81 |
| DISP | -3.88 | -3.99 | -8.94 | -9.67 | -10.08 | -8.19 |
|  | **3+1** | **3+2** | **3+3** | **4+1** | **4+2** | **5+1** |
| ELC | -169.55 | -167.80 | -192.27 | -178.90 | -198.20 | -189.18 |
| EX | -81.40 | -76.58 | -80.98 | -79.88 | -86.00 | -79.57 |
| REP | 285.69 | 261.72 | 282.42 | 279.35 | 301.09 | 275.48 |
| POL | -134.12 | -126.54 | -134.17 | -135.03 | -145.56 | -141.86 |
| DISP | -13.48 | -11.27 | -12.14 | -14.91 | -16.67 | -17.80 |

**Supplementary Table 9(C).** Contribution of energy components (kcal/mol) such as electrostatic (ELC), exchange (EX), repulsion (REP), polarization (POL), dispersion (DISP), and into the interaction energy of doublet Sc^2+^(OH_2_)_n_; n=1-6 complexes obtained using LMO-EDA scheme at B3LYP/6-311G**//MP2/6-31G* for Hydrogen (H) and Oxygen (O) and B3LYP/cc-pVTZ//MP2/6-31G* for metal ion.

| **Energy components** | **1+0** | **2+0** | **3+0** | **4+0** | **5+0** | **6+0** |
| --- | --- | --- | --- | --- | --- | --- |
| ELC | -56.52 | -114.62 | -168.72 | -198.94 | -227.64 | -236.48 |
| EX | -6.32 | -15.73 | -27.08 | -30.00 | -30.76 | -18.82 |
| REP | 32.15 | 78.26 | 131.47 | 139.35 | 150.98 | 104.82 |
| POL | -36.60 | -73.14 | -107.14 | -130.27 | -145.49 | -132.76 |
| DISP | -4.98 | -10.64 | -17.22 | -18.29 | -22.56 | -24.05 |
|  | **1+1** | **1+2** | **2+1** | **2+2** | **2+3** | **2+4** |
| ELC | -103.65 | -134.13 | -157.52 | -181.82 | -206.75 | -233.66 |
| EX | -16.25 | -16.00 | -24.61 | -22.15 | -21.32 | -29.29 |
| REP | 71.42 | 77.12 | 112.96 | 108.29 | 107.80 | 132.84 |
| POL | -61.19 | -68.04 | -91.26 | -94.11 | -95.96 | -109.17 |
| DISP | -6.43 | -7.43 | -11.95 | -12.83 | -12.82 | -12.91 |
|  | **3+1** | **3+2** | **3+3** | **4+1** | **4+2** | **5+1** |
| ELC | -201.71 | -222.21 | -258.66 | -238.86 | -252.22 | -250.65 |
| EX | -26.65 | -31.39 | -38.39 | -45.05 | -28.70 | -28.03 |
| REP | 135.69 | 146.42 | 178.18 | 201.35 | 146.13 | 142.90 |
| POL | -110.74 | -124.21 | -142.78 | -164.63 | -126.27 | -141.08 |
| DISP | -17.98 | -17.37 | -16.09 | -18.29 | -21.90 | -23.93 |

**Supplementary Table 9(D).** Contribution of energy components (kcal/mol) such as electrostatic (ELC), exchange (EX), repulsion (REP), polarization (POL), dispersion (DISP), and into the interaction energy of singlet Sc^3+^(OH_2_)_n_; n=1-6 complexes obtained using LMO-EDA scheme at B3LYP/6-311G**//MP2/6-31G* for Hydrogen (H) and Oxygen (O) and B3LYP/cc-pVTZ//MP2/6-31G* for metal ion.

| **Energy components** | **1+0** | **2+0** | **3+0** | **4+0** | **5+0** | **6+0** |
| --- | --- | --- | --- | --- | --- | --- |
| ELC | -65.55 | -106.82 | -217.75 | -208.98 | -276.17 | -262.63 |
| EX | -22.20 | -32.84 | -115.21 | -95.37 | -141.68 | -123.27 |
| REP | 95.62 | 138.46 | 428.86 | 355.72 | 529.89 | 456.39 |
| POL | -37.22 | -57.34 | -159.85 | -129.74 | -214.56 | -194.96 |
| DISP | -6.72 | -12.68 | -20.58 | -26.28 | -29.63 | -32.63 |
|  | **1+1** | **1+2** | **2+1** | **2+2** | **2+3** | **2+4** |
| ELC | -165.04 | -217.72 | -232.52 | -249.99 | -284.13 | -314.11 |
| EX | -18.83 | -23.81 | -23.45 | -15.77 | -18.13 | -19.03 |
| REP | 116.10 | 145.10 | 148.73 | 102.92 | 116.60 | 121.95 |
| POL | -159.36 | -187.20 | -215.88 | -196.63 | -210.83 | -219.92 |
| DISP | -11.27 | -12.30 | -17.73 | -15.31 | -15.81 | -16.06 |
|  | **3+1** | **3+2** | **3+3** | **4+1** | **4+2** | **5+1** |
| ELC | -289.15 | -314.27 | -334.03 | -244.83 | -353.28 | -261.17 |
| EX | -26.40 | -24.25 | -20.33 | -111.08 | -25.94 | -118.14 |
| REP | 170.95 | 157.89 | 134.07 | 415.11 | 171.66 | 439.05 |
| POL | -260.48 | -259.09 | -254.31 | -157.46 | -296.43 | -180.20 |
| DISP | -23.07 | -22.24 | -20.04 | -26.81 | -26.14 | -30.17 |

**Supplementary Table 10.** Reaction energy (ΔE_R_) for the reaction Sc^+^ + OH_2_🡪Sc^+^O + H_2_ + ΔE, and sequential binding energy (${\Delta E}_{\mathrm{seq}}$) of ground state Sc^+^OH_2_ complex. All values are reported in kcal/mol.

| **Methods** | **ΔE_seq_** | **ΔE_R_** |
| --- | --- | --- |
| BP86/6-311G** | -38.40 | 57.59 |
| BP86/aug-cc-pVTZ | -33.14 | 52.13 |
| B97D/6-311G** | -34.51 | 66.43 |
| B97D/aug-cc-pVTZ | -29.98 | 60.77 |
| B3LYP-D3/6-311G** | -40.73 | 42.15 |
| B3LYP-D3/aug-cc-pVTZ | -35.28 | 38.20 |
